# The Phylogenetically-Related Pattern Recognition Receptors EFR and XA21 Recruit Similar Immune Signaling Components in Monocots and Dicots

**DOI:** 10.1371/journal.ppat.1004602

**Published:** 2015-01-21

**Authors:** Nicholas Holton, Vladimir Nekrasov, Pamela C. Ronald, Cyril Zipfel

**Affiliations:** 1 The Sainsbury Laboratory, Norwich Research Park, Norwich, United Kingdom; 2 Department of Plant Pathology and the Genome Center, University of California, Davis, Davis, California, United States of America; University of California Riverside, UNITED STATES

## Abstract

During plant immunity, surface-localized pattern recognition receptors (PRRs) recognize pathogen-associated molecular patterns (PAMPs). The transfer of PRRs between plant species is a promising strategy for engineering broad-spectrum disease resistance. Thus, there is a great interest in understanding the mechanisms of PRR-mediated resistance across different plant species. Two well-characterized plant PRRs are the leucine-rich repeat receptor kinases (LRR-RKs) EFR and XA21 from *Arabidopsis thaliana* (Arabidopsis) and rice, respectively. Interestingly, despite being evolutionary distant, EFR and XA21 are phylogenetically closely related and are both members of the sub-family XII of LRR-RKs that contains numerous potential PRRs. Here, we compared the ability of these related PRRs to engage immune signaling across the monocots-dicots taxonomic divide. Using chimera between Arabidopsis EFR and rice XA21, we show that the kinase domain of the rice XA21 is functional in triggering elf18-induced signaling and quantitative immunity to the bacteria *Pseudomonas syringae* pv. *tomato* (*Pto*) DC3000 and *Agrobacterium tumefaciens* in Arabidopsis. Furthermore, the EFR:XA21 chimera associates dynamically in a ligand-dependent manner with known components of the EFR complex. Conversely, EFR associates with Arabidopsis orthologues of rice XA21-interacting proteins, which appear to be involved in EFR-mediated signaling and immunity in Arabidopsis. Our work indicates the overall functional conservation of immune components acting downstream of distinct LRR-RK-type PRRs between monocots and dicots.

## Author Summary

Pests and diseases cause significant agricultural losses. Plants recognize pathogen-derived molecules via plasma membrane-localized immune receptors (called pattern recognition receptors or PRRs), resulting in pathogen resistance. In recent years, the transfer of PRRs across plant species has emerged as a promising biotechnological approach to improve crop disease resistance. Successful transfers of PRRs suggest that immune signaling components are conserved across plant species. In this study, we demonstrate that the PRR XA21 from the monocot plant rice is functional in the dicot plant *Arabidopsis thaliana* (Arabidopsis) and that it confers quantitatively enhanced resistance to bacteria. Furthermore, we show that the rice XA21 and the Arabidopsis EFR, which are evolutionary-distant but phylogenetically closely related, recruit similar signaling components for their function, revealing an overall conservation of immune pathways across monocots and dicots. These findings demonstrate evolutionary conservation of downstream signaling from PRRs and indicate that transfer of PRRs is possible between different plant families, but also between monocots and dicots.

## Introduction

The first line of innate immunity in plants is conferred by surface-localized pattern recognition receptors (PRRs) that recognize microbial patterns, referred to as pathogen-associated molecular patterns (PAMPs). PRR-triggered immunity (PTI) is involved in basal resistance to adapted pathogens and in non-host resistance to non-adapted pathogens. Furthermore, PAMP recognition triggers both local and systemic immune responses in plants.

Plant PRRs are either receptor kinases (RKs) or receptor-like proteins (RLPs). RKs are modular proteins with an extracellular domain potentially involved in ligand binding, a single pass transmembrane domain and an intracellular kinase domain that relays downstream signaling. In contrast to RKs, RLPs do not possess any kinase domain and only have a short cytoplasmic tail that lacks any obvious signaling motifs. Thus, RLPs most likely form heteromeric complexes with RKs or other cytoplasmic kinases to transduce signaling upon ligand perception. RKs represent one the largest plant protein families, with leucine-rich repeat (LRR)-type RKs being the largest group of RKs [1]. For example, the Arabidopsis and rice genomes encode >600 and >900 RKs of which >220 and >290 are LRR-RKs, respectively [2–5]. Plant RKs are evolutionary related and have mostly evolved through duplication events leading to the expansion of the family [3,5–8].

The first LRR-RK implicated in plant immunity was rice XA21, which confers resistance to the bacterium *Xanthomonas oryzae* pv. *oryzae* (*Xoo*, the causal agent of bacterial blight) [9]. The ligand for XA21 is still unknown [10]. XA21 belongs to the sub-family XII of LRR-RKs (LRR-XII), which is one of the most expanded sub-families of LRR-RKs in rice with >150 members. This family also contains numerous members in other sequence genomes, such as poplar (*Populus trichocarpa*) with >40 members [11], tomato (*Solanum lycopersicum*) with >50 members [12] and grapevine (*Vitis vinifera*) with >34 members [4]. In contrast, *Arabidopsis thaliana* (hereafter referred to as Arabidopsis) only contains 10 sub-family LRR-XII members [8]. Despite its relatively small size, the Arabidopsis LRR-XII sub-family contains FLS2 and EFR that recognize bacterial flagellin (or the derived peptide flg22) and elongation factor TU (EF-Tu) (or the derived peptide elf18), respectively [13,14], and which are amongst the best-characterized PRRs in plants. Notably, XA21, FLS2 and EFR are non-RD kinases (where RD refer to conserved Arg and Asp residues in the kinase subdomain VIb), a feature correlated with a function in innate immunity across kingdoms [15,16]. The presence of non-RD kinase domains in most members of the sub-family LRR-XII in diverse plant species suggests that this sub-family encodes mostly for PRRs or PRR-associated RKs [16].

The transfer of PRRs between plant species is a promising approach for engineering broad-spectrum disease resistance that may be durable [17,18]. Such transfer has already been used in conventional breeding programs to generate hybrids with PRR-mediated resistance from wild relatives as single or pyramided loci [19]. For example, XA21 was originally introgressed from a wild species of rice (*Oryza longistaminata*) into cultivated rice (*Oryza sativa*) [9,20]. However, these approaches are restricted by inter-species incompatibilities. In recent years, several reports have described the successful transgenic transfer of PRRs into phylogenetically diverse species leading to improved disease resistance. For example, transgenic expression of Arabidopsis EFR into the distantly related solanaceous dicot species *Nicotiana benthamiana* and tomato provides broad-spectrum resistance to bacteria belonging to different genera [21]. Conversely, transfer of the tomato LRR-RLP Ve1 into Arabidopsis confers resistance to different Verticillium species [22]. Also, rice XA21 has been introduced into the monocot banana (*Musa sp*.) and confers resistance to Banana Xanthomonas wilt caused by *X. campestris* pv. *musacearum* [23]. Furthermore, transgenic expression of rice XA21 in the dicot species tomato and sweet orange (*Citrus x sinensis*) results in improved resistance to bacterial wilt caused by *Ralstonia solanacearum* and citrus canker caused by *X. axonopodis* pv. *citri*, respectively [24,25]. Importantly, these examples suggest that PRR-mediated signaling pathways are conserved across plant species, families, and even clades, and that the transferred PRRs can recruit biochemically these potentially conserved immune components.

A number of genetic and biochemical studies have identified components regulating PRR biogenesis, accumulation and downstream signaling in Arabidopsis and rice [26–28]. Consistent with their close phylogenetic relationship [2–5], both EFR and XA21 heavily rely on evolutionary conserved endoplasmic reticulum-quality control (ER-QC) components for proper folding and accumulation [29–35]. These requirements may illustrate the relatively younger age of EFR and XA21 in evolutionary terms, since these PRRs seem to have evolved recently in *Brassicaceae* and in wild rice species, respectively. In contrast, FLS2, which is more distantly related to XA21 than is EFR, is conserved in all vascular plants [36] and does not show similar genetic and biochemical ER-QC requirements for its biogenesis.

In terms of downstream signaling, FLS2 and EFR engage largely overlapping components leading to congruent immune outputs, including transcriptional changes [13,37]. In summary, they form ligand-dependent heteromers with the LRR-RK BAK1/SERK3 (and related SERK proteins) that acts as co-receptor and positive regulator [38–41]. Receptor-like cytoplasmic kinases (RLCKs), such as BIK1 and PBL1, are direct substrates of the FLS2/EFR-BAK1 complexes and their ligand-induced phosphorylation lead to activation of specific immune outputs [42–44]. Recently, it has been shown that activated BIK1 directly phosphorylates the NADPH oxidase RBOHD to trigger an apoplastic burst of reactive oxygen species (ROS), one of the hallmark early PTI responses [45,46]. Other immune responses triggered upon flg22 or elf18 perception include activation of mitogen-activated protein (MAP) and calcium-dependent protein kinases (MAPKs and CDPKs), callose deposition and expression of immune-related genes [36]. Prior ligand-binding, the formation of the FLS2(/EFR)-BAK1 is inhibited by the BAK1-interacting LRR-RK BIR2 [47]. Upon heteromerization with BAK1, the BAK1-associated E3 ligases PUB12 and PUB13 are transferred to FLS2 (and potentially EFR) to ubiquitinate the ligand-bound PRR, targeting it to degradation [48]. Also, ligand-bound FLS2 is endocytosed [49]. These two mechanisms are most likely required to enable replenishment of the plasma membrane with ligand-free PRRs.

Signaling components acting downstream of XA21 have been mostly identified through interaction studies. In the absence of ligand, XA21 is kept inactive by the ATPase XB24 that triggers XA21 auto-phosphorylation, keeping its kinase inactive [50]. After activation and XB24 dissociation, XA21 activity is inhibited by the protein phosphatase 2C (PP2C) XB15 through dephosphorylation [51]. The E3 ligase XB3 and the plant-specific ankyrin-repeat (PANK) protein XB25 are XA21-interacting proteins that are required for its accumulation [52,53]. Mechanisms that positively regulate XA21-mediated early immune signaling are mostly undefined. Only very recently, it was shown that the rice SERK protein OsSERK2 constitutively associates with XA21 and positively regulates XA21-mediated immunity to *Xoo* [54].

Surprisingly, despite belonging to the same sub-family of LRR-RKs, a comparison of the currently known molecular mechanisms underlying immune signaling triggered by FLS2/EFR and XA21 in Arabidopsis and rice, respectively, reveals very little similarity. The lack of a proven ligand for XA21 [10] and the lack of robust assays for XA21-mediated immune signaling have hindered the study of dynamic immune signaling upon XA21 activation.

In this study, we directly compared the ability of two evolutionary-distant, yet phylogenetically closely related PRRs to engage immune signaling in a single plant species (*Arabidopsis thaliana*). Using chimera between Arabidopsis EFR and rice XA21, we show that the kinase domain of the rice XA21 is functional in Arabidopsis to induce signaling and quantitative immunity to the bacterium *Pseudomonas syringae* pv. *tomato* (*Pto*) DC3000 and *Agrobacterium tumefaciens*. In addition, our results suggest that the XA21 extracellular domain may perceive a ligand derived from *Pto* DC3000. The EFR:XA21 chimera associates dynamically in a ligand-dependent manner with known components of the EFR complex. Conversely, EFR associates with Arabidopsis orthologues of rice XA21-interacting proteins, which appear to be involved in EFR-mediated signaling and immunity in Arabiodpsis. Our work indicates the overall functional conservation of immune components acting downstream of distinct LRR-RK-type PRRs between monocots and dicots.

## Results/Discussion

### The XA21 intracellular domain confers immune signaling in dicots

**Figure 1:**
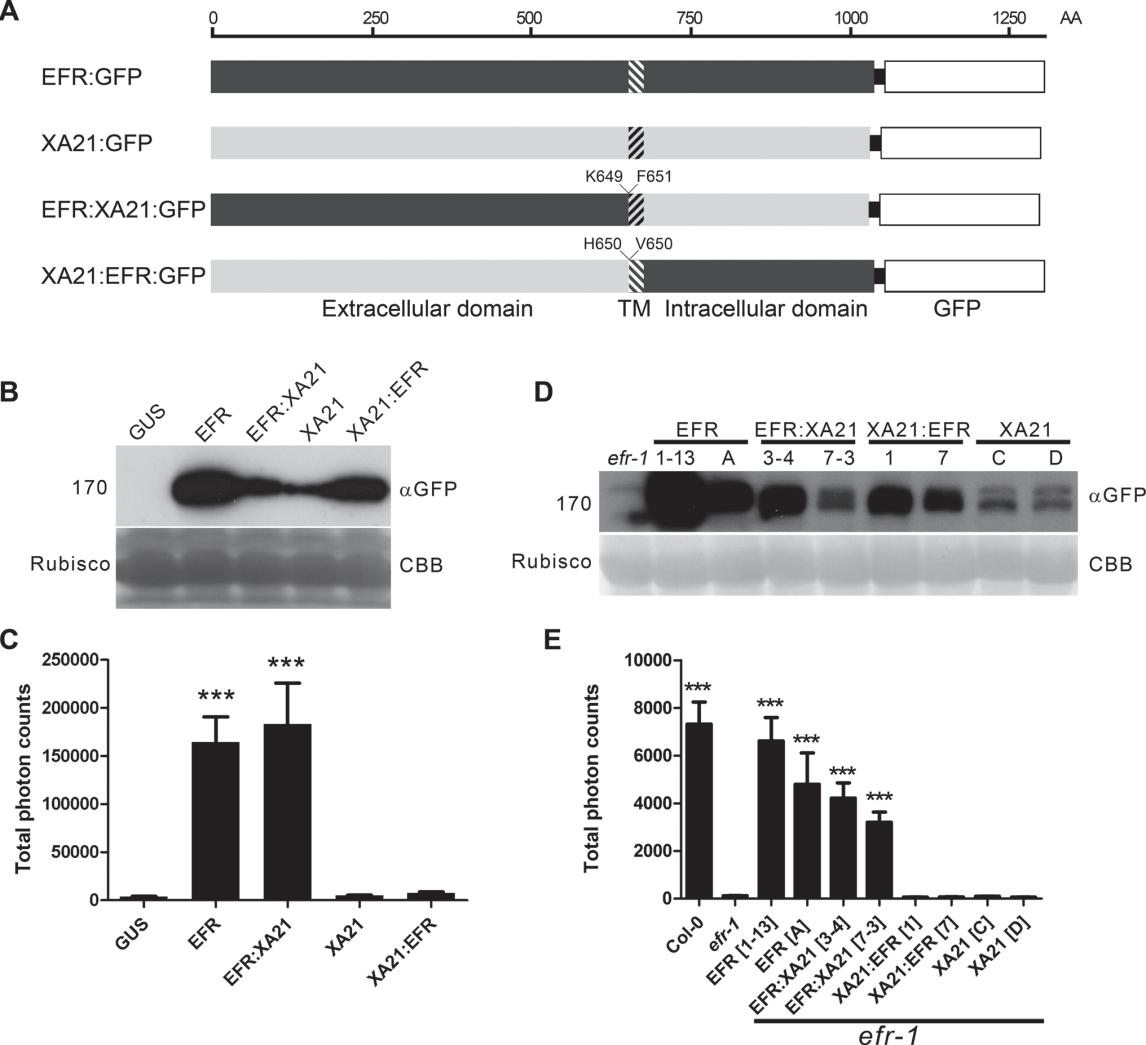
The EFR:XA21 chimera confers immune signaling in *N. benthamiana* and *A. thaliana*. A. Schematic representation of chimeric PRRs generated in this study. Hatched shading indicates the transmembrane region. Amino acids at the boundary of the protein fusion are indicated. B. Accumulation of GUS, EFR:GFP, EFR:XA21:GFP; XA21:GFP; XA21:EFR:GFP proteins transiently expressed under the control of the *35S* promoter in *N. benthamiana*. Protein was extracted at the same time point after infection as the ROS assays presented in (C) were performed. C. Elf18-induced ROS production following transient expression of *GUS, EFR:GFP, EFR:XA21:GFP, XA21:GFP* or *XA21:EFR:GFP* transiently expressed under the control of the *35S* promoter in *N. benthamiana*. ROS production is represented as the total of photons emitted during 40 min after treatment with 100 nM elf18. Values are averages ±SE (*n* = 12). D. Accumulation of GFP fusions in *A. thaliana efr-1* and *efr-1* transgenic homozygous lines stably expressing *EFR:GFP, EFR:XA21:GFP, XA21:EFR:GFP* or *XA21:GFP* under the control of the *35S* promoter. Two independent lines are shown; the same as those used for ROS induction in (E). Western blots were performed with HRP-conjugated anti-GFP antibodies. Membranes were stained with Coomassie brilliant blue (CBB) to document equal protein loading. E. Elf18-induced ROS production in *A. thaliana* Col-0, *efr-1* and *efr-1* transgenic homozygous lines stably expressing *EFR:GFP, EFR:XA21:GFP, XA21:EFR:GFP* or *XA21:GFP* under the control of the *35S* promoter. ROS production is represented as the total of photons emitted during 40 min after treatment with 100 nM elf18. Values are averages ±SE (*n* = 12). Experiments performed three times with similar results. Asterisks indicate significant differences from *efr-1*; *p* < 0.001 (Student’s t-test).

To study the functionality of XA21 in dicots, we constructed chimeric receptors between EFR and XA21, and compared them to full-length EFR and XA21 proteins. Chimeras (EFR:XA21 and XA21:EFR) were produced by fusion of the extracellular domain with the transmembrane/intracellular domain, at the external boundary of the transmembrane domain (Fig. 1A). Previously Albert *et al*. [55] examined the functionality of EFR:FLS2 chimeras and found fusion at an equivalent position was functional. All proteins were C-terminally tagged with green fluorescent protein (GFP) to check protein accumulation. Because the XA21 ligand is currently unknown, the availability of the EFR:XA21 chimera allows assessment of the activation of the XA21 intracellular domain in response to elf18 binding to the EFR extracellular domain.

We first tested the functionality of these receptors by expressing them transiently in *Nicotiana benthamiana*, which lacks endogenous *EFR* and thus does not perceive elf18 [13,21]. Immunoblot analysis indicated that all proteins accumulated (Fig. 1B) and localized predominantly to the plasma membrane (S1 Fig.). Activation of these receptors was then tested by analyzing the ability to confer an elf18-induced ROS burst. Both EFR and EFR:XA21 expression allowed the similar production of ROS in response to elf18 (Fig. 1C). However, the expression of neither XA21 or XA21:EFR led to production of ROS greater than that seen when the enzyme β-glucuronidase (GUS; used as a transformation control) was expressed (Fig. 1C).

We then sought to confirm these results upon stable expression in Arabidopsis. To this end, we generated transgenic lines in a null *efr-1* mutant background with the constructs. We selected two independent homozygous lines that properly accumulated the tagged receptors (Fig. 1D) for further analysis. As observed upon transient expression in *N. benthamiana*, only EFR and EFR:XA21 conferred elf18 responsiveness (Fig. 1E). Notably, the total amount of ROS generated correlated with protein accumulation (Fig. 1D).

To further characterize the function of the XA21 cytoplasmic domain, we compared quantitatively the ability of EFR and EFR:XA21 to induce elf18-induced marker gene expression when expressed stably in Arabidopsis. Notably, the expression levels of *PR1, FRK1, PHI1* and *NHL10* following efl18 treatment was comparable in Col-0, EFR and EFR:XA21 plants (S2 Fig.). Importantly, the basal expression of these immune marker genes was not affected in the transgenic lines expressing EFR or EFR:XA21 (S2 Fig.), indicating that these plants do not show any sign of constitutive immune responses.

Together, these data showed that the chimeric receptor EFR:XA21 is capable of responding to elf18, demonstrating that the XA21 cytoplasmic domain can substitute for the EFR cytoplasmic domain to induce immune outputs. However, the XA21 extracellular domain did not confer elf18 responsiveness, indicating that XA21 does not bind elf18.

### Expression of XA21 and the EFR:XA21 chimera in Arabidopsis increases immunity to pathogenic bacteria

**Figure 2:**
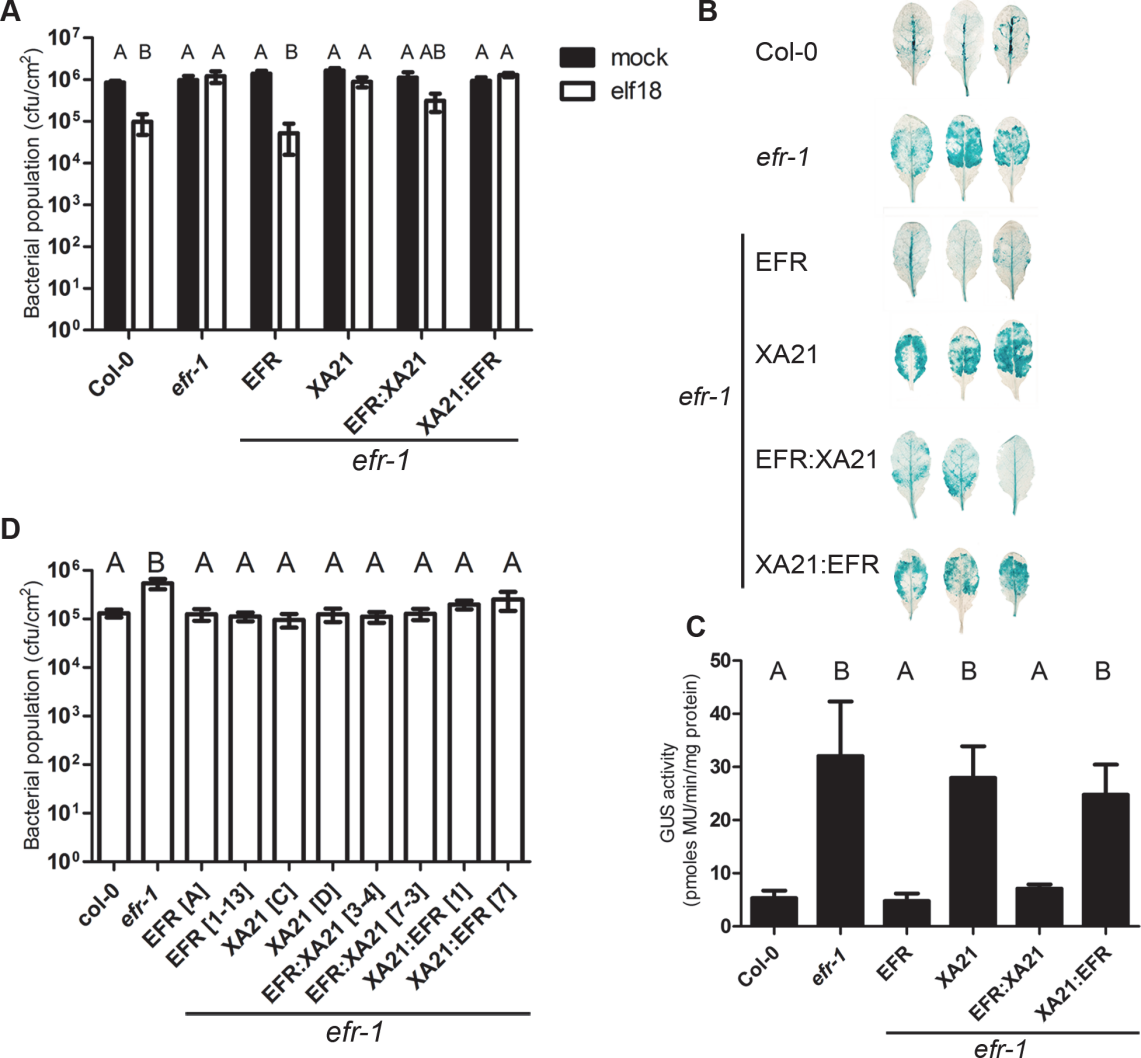
Bacterial resistance conferred by EFR, XA21 and chimeric receptors in *A. thaliana*. A. Elf18-induced resistance in *A. thaliana* Col-0, *efr-1* and *efr-1* transgenic homozygous lines stably expressing *EFR:GFP, EFR:XA21:GFP, XA21:GFP* or *XA21:EFR:GFP* under the control of the *35S* promoter. Plants were pre-treated with mock (water) or 1 μM elf18 for 24 h before infection with *Pto* DC3000 (OD_600_ = 0.0002). Bacterial populations were scored at 2 dpi. Values are averages ±SE (*n* = 4). Infections were repeated four times with similar results. B. *Agrobacterium*-mediated transient expression of a *35S::GUS* transgene in *A. thaliana* Col-0, *efr-1* and *efr-1* transgenic homozygous lines stably expressing *EFR:GFP, XA21:GFP, EFR:XA21:GFP* or *XA21:EFR:GFP* under the control of the *35S* promoter. Leaves were stained for GUS activity at 3 dpi. Leaves shown are representative of the variation in GUS staining observed. The experiment was repeated four times with similar results. C. Fluorimetric quantification of GUS activity. Values are averages ±SE (*n* = 6). The experiment was repeated three times with similar results. D. Susceptibility of *A. thaliana* Col-0, *efr-1* and *efr-1* transgenic homozygous lines stably expressing *EFR:GFP, XA21:GFP, EFR:XA21:GFP*, or *XA21:EFR:GFP* under the control of the *35S* promoter to *Pto* DC3000 *COR^−^* (OD_600_ = 0.2; spray inoculation). Bacterial populations were scored at 3 dpi. Values are averages ±SE (*n* = 4). Different letters indicate significantly different values at *p* <0.05 (one-way ANOVA). The experiment was repeated 5 times with similar results.

Next, we determined if the signaling outputs triggered by the XA21 intracellular domain could lead to increased immunity to pathogens in Arabidopsis. Pre-treatment of Arabidopsis plants with elf18 restricts growth of the plant pathogenic bacterium *Pseudomonas syringae* pv. *tomato* (*Pto*) DC3000 by about 1 log, which is lost in the null *efr-1* mutant (Zipfel et al., 2006). The transgenic lines expressing EFR produced the same level of elf18-induced resistance than wild-type Col-0 plants, while the EFR:XA21 lines produced a lower level of elf18-induced resistance (Fig. 2A). This is on contrast to the transgenic lines expressing XA21 and XA21:EFR, which were insensitive to elf18 as observed in the parental *efr-1* line (Fig. 2A).

Letters indicate statistically significant differences from Tukey’s HSD mean separation (*p* < 0.05).

EFR expression also contributes to resistance to *Agrobacterium tumefaciens* by recognising the endogenous EF-Tu [13,21], which can be measured in an *Agrobacterium*-mediated transient expression assay using an intronic GUS transgene followed by a colorimetric or fluorometric assay to quantify GUS enzymatic activity. Leaves of the null *efr-1* mutants allow more GUS expression than wild-type Col-0 leaves (Fig. 2B, C), as previously observed [13]. The transgenic lines expressing EFR or EFR:XA21 complemented the *efr-1* phenotype and exhibited low GUS expression to a level comparable to Col-0 (Fig. 2B, C). In contrast, levels of GUS expression observed in the XA21 and XA21:EFR lines were similar to those observed in *efr-1* (Fig. 2B, C). Thus, the presence of the intracellular domain of XA21 enabled transmission of elf18 or EF-Tu perception by the EFR extracellular domain into an immune output leading to improved pathogen resistance.

We were also interested to test the potential contribution of the XA21 intracellular domain in the basal resistance to *Pto* DC3000. For this, we used the hypo-virulent strains *Pto* DC3000 *COR^−^* after spray-inoculation, an assay that has been used previously to reveal the contribution of FLS2 and EFR to this bacterium [33,56,57]. This strain is affected in biosynthesis of the toxin coronatine, which is a jasmonic acid mimic involved in the suppression of pre- and post-invasive immunities [58]. As previously reported [56], *efr-1* plants are more susceptible to *Pto* DC3000 *COR^−^* (Fig. 2D). Consistent with our previous results (Figs. 1 C, E and 2 A–C), expression of EFR or EFR:XA21 was capable of complementing *efr-1* (Fig. 2D). Surprisingly, expression of XA21 or XA21:EFR in two independently transformed lines also resulted in improved resistance to *Pto* DC3000 *COR^−^* in the *efr-1* background (Fig. 2D). This effect cannot be caused by recognition of the elf18 epitope derived from *Pto* EF-Tu, as these receptors did not confer elf18-dependent responsiveness in previous assays (Figs. 1 C, E and 2 A–C). These data suggest that the XA21 ectodomain may be able to perceive a ligand derived from *Pto* DC3000.

It cannot be excluded that ectopic over-expression of XA21 or XA21:EFR may result in some degree of auto-activation leading to a ligand-independent enhanced disease resistance. To test if these lines show signs of constitutive immune responses, basal *PR1* expression (as a marker for SA perception/signaling) and MAPK activity were measured (S3 Fig.). No significant basal *PR1* expression or MAPK activity was observed, suggesting that immune responses are not auto-activated in these lines. Expression of XA21 or XA21:EFR may enable the recognition of bacteria expressing the XA21 ligand in the phyllosphere or rhizosphere that would ‘prime’ immune responses. However, we did not observe any necrosis or growth defects in these lines, and they were also not more resistant to Agrobacterium or *Pto* DC3000 upon infiltration (Fig. 2A–C), which would be expected if these hypotheses were correct.

XA21-mediated resistance in rice relies on the expression of several components genetically identified in *X. oryzae* pv. *oryzae*, namely: the predicted type I secretion components *raxA, raxB* and *raxC*; *raxP, raxQ* and *raxST*, involved in the regulation of 3′-phosphoadenosine 5′-phosphosulfate generation and sulfation; and *raxR/H*, which controls *hrp* gene expression [59–61]. *Pto* DC3000 possesses many of these components, except for *raxST* [62]. While these components are important for generating a functional XA21 ligand in *Xoo*, it is not known whether these bacterial genes are required for production of a functional XA21 ligand in other bacterial species. Notably, transgenic XA21 expression in tomato provides resistance to *Ralstonia solanacearum* [24], which contains a *raxST* homologue unlike *Pto*, which lacks raxST These data and the results presented here suggest that XA21 may recognize a ligand present in many bacterial pathogens and that its function is not restricted to recognition of *Xanthomonas* species. The future identification of the XA21 ligand(s) is required to address these questions. The availability of the Arabidopsis XA21:EFR line generated in this study and of well-characterized quantitative early immune outputs triggered by the EFR intracellular domain may be helpful to tackle the challenge of identifying the elusive XA21 ligand.

### The EFR:XA21 chimera associates dynamically with BAK1 and BIK1 in a ligand-dependent manner

The functionality of the XA21 intracellular domain in conferring immune signaling and immunity upon elf18 perception by the EFR extracellular domain (Figs. 1 and 2) suggests that the EFR:XA21 chimera recruit similar downstream signaling components as EFR.

**Figure 3:**
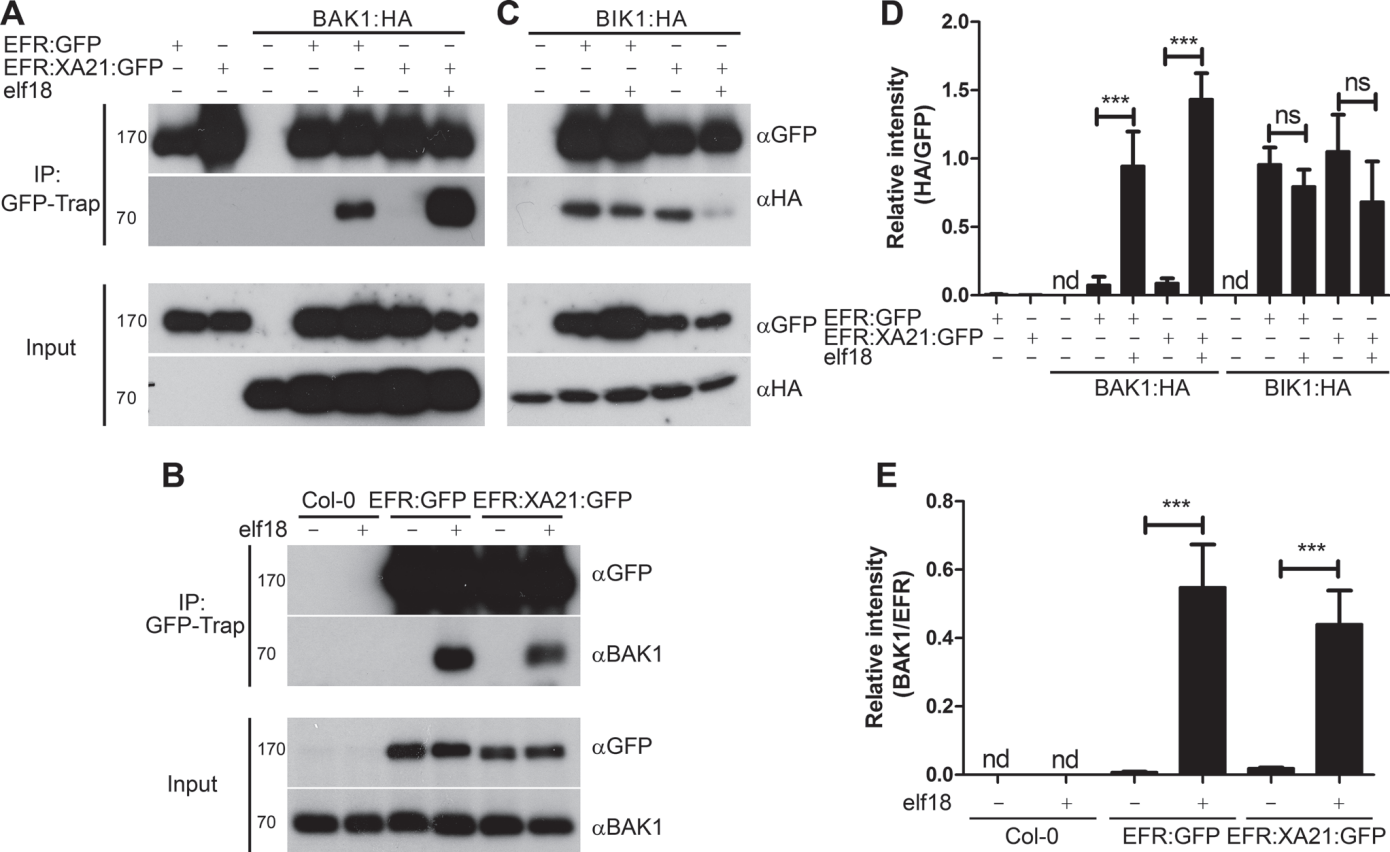
Dynamic association of EFR and the EFR:XA21 chimera with BAK1 and BIK1 *in planta*. A. Co-immunoprecipitation of transiently-expressed EFR:GFP and EFR:XA21:GFP with BAK1:HA in *N. benthamiana*. Leaves were treated with 100 nM elf18 (+) or mock treated with water (−) for 10 min. Immunoprecipitation (IP) was performed with GFP-Trap agarose beads. Western blots were performed with HRP-conjugated anti-GFP and anti-HA antibodies. Experiments were performed at least twice with similar results. B. Co-immunoprecipitation with BAK1 in *A. thaliana* Col-0 and *efr-1* transgenic homozygous lines stably expressing *EFR:GFP* or *EFR:XA21:GFP* under the control of the *35S* promoter. Leaves were treated with 100 nM elf18 (+) or mock treated with water (−) for 10 min. Immunoprecipitation (IP) was performed with GFP-Trap agarose beads. Western blots were performed with HRP-conjugated anti-GFP, anti-HA or anti-BAK1 antibodies. Experiments were performed at least twice with similar results. C. Co-immunoprecipitation of transiently-expressed EFR:GFP and EFR:XA21:GFP with BIK1:HA in *N. benthamiana*. Leaves were treated with 100 nM elf18 (+) or mock treated with water (−) for 10 min. Immunoprecipitation (IP) was performed with GFP-Trap agarose beads. Western blots were performed with HRP-conjugated anti-GFP and anti-HA antibodies. Experiments were performed at least twice with similar results. D, E. Quantitation of data presented in A and C. Values are the average ratio of BAK:HA or BIK:HA to EFR:GFP ±SE (*n* = 3). Asterisks indicate statistical significance of mock compared to elf18 treated, *p* < 0.001 (Student’s t-test). ns is not significant at *p* <0.05. nd is not determined.

BAK1 associates dynamically with FLS2 and EFR in a ligand-dependent manner [39–41,63]. A recent structural study demonstrated that flg22 acts as ‘molecular glue’ between the flg22-bound FLS2 and BAK1 enabling the quasi-instantaneous complex formation between the two proteins [38]. Thus, BAK1 acts as a co-receptor. Notably, a similar mechanism has been observed for the ligand-induced complex formation between the brassinosteroid receptor BRI1 and its co-receptors BAK1 and SERK1 [64,65]. To investigate if the EFR:XA21 chimera could also associated with BAK1 in a ligand-dependent manner, GFP-tagged EFR and EFR:XA21 were transiently co-expressed with hemagglutinin (HA)-tagged BAK1 in *N. benthamiana* followed by treatment with mock or elf18 for 10 min. Immunoprecipitation of EFR or EFR:XA21 using GFP-Trap beads demonstrated that BAK1 only co-precipitated following elf18 treatment (Fig. 3A,D). In addition, ligand-dependent association of native BAK1 was also observed in transgenic Arabidopsis plants expressing EFR or EFR:XA21 (Fig. 3B). To determine whether the XA21 cytoplasmic domain is required for the interaction with BAK1, we created truncated chimeras that contain only the extracellular and transmembrane domains (including a short positively charged region of the intracellular juxtamembrane). Like the full-length receptor, interaction with BAK1 was observed only following elf18 treatment (S4 Fig.). However this interaction is not sufficient to elicit elf18-induced ROS burst. These data indicate that the extracellular domain is sufficient for BAK1 interaction, as previously shown in the case of the association between FLS2 and BAK1 [38].

Interestingly, a rice BAK1 orthologue, OsSERK2, was very recently shown to associate constitutively with XA21 and OsFLS2 [54], suggesting distinct biochemical mechanisms of association with SERK proteins between monocots and dicots. Importantly, it remains to be tested if the association between XA21-OsSERK2 and OsFLS2-OsSERK2 would be stronger upon ligand treatment. Our data suggest that the constitutive interaction of PRRs with OsSERK2 observed in rice is not due to a property of the XA21 intracellular domain. Such constitutive interaction must therefore be dependent on OsSERK2 or the extracellular domains of XA21 and OsFLS2. It is also possible that components that control the interactions between SERKs and RKs plays distinct functions between monocots and dicots, or that they are absent in monocots. For example, Arabidopsis BIR2 has been recently demonstrated to interact with BAK1 and prevent or reduce its association with FLS2 [47]. While homologues of BIR2 are present in rice, it is not known if they associate with OsSERK2 and control its interaction with PRRs.

The RLCKs BIK1 and related PBL proteins are direct substrates of EFR and other PRRs [43,44,66]. Transient co-expression of GFP-tagged EFR or EFR:XA21 with HA-tagged BIK1 was performed to determine the interaction with BIK1. BIK1 constitutively associated with both EFR and EFR:XA21 (Fig. 3C,D). Following elf18 treatment, we observed partial dissociation of these complexes, similar to that previously reported between EFR and BIK1 in Arabidopsis [43]. These results illustrate that the XA21 intracellular domain can dynamically associate with BIK1.

BIK1 homologues exist in rice and other monocots. One of these, OsRLCK185, interacts with the LysM-RK CERK1 involved in chitin perception and is involved in chitin-mediated signaling [67]. Notably, in Arabidopsis, BIK1 is also involved in chitin signaling [43], suggesting that BIK1 is a convergent component of both BAK1-dependent and BAK1-independent PRR-triggered signaling pathways. Whether XA21 interacts with OsRLCK185 remains to be tested. Together, these data nevertheless already indicate an overall conservation in the molecular mechanisms underlying the BIK1-PRR interactions between monocots and dicots.

### EFR associates dynamically with Arabidopsis orthologues of XA21-interacting proteins in a ligand-dependent manner

The functionality of the EFR:XA21 and XA21 proteins in Arabidopsis, and the association of EFR:XA21 with known EFR signaling components suggests a large degree of conservation between XA21 and EFR signaling in monocots and dicots, respectively. Several XA21-interacting proteins have been identified in rice [26], but interestingly there is currently no evidence that their corresponding orthologues in dicots may also be involved in PRR signaling. We directly investigated whether Arabidopsis orthologues of some of these XA21 signaling components, namely XB15 and XB24, are involved in EFR signaling.

**Figure 4:**
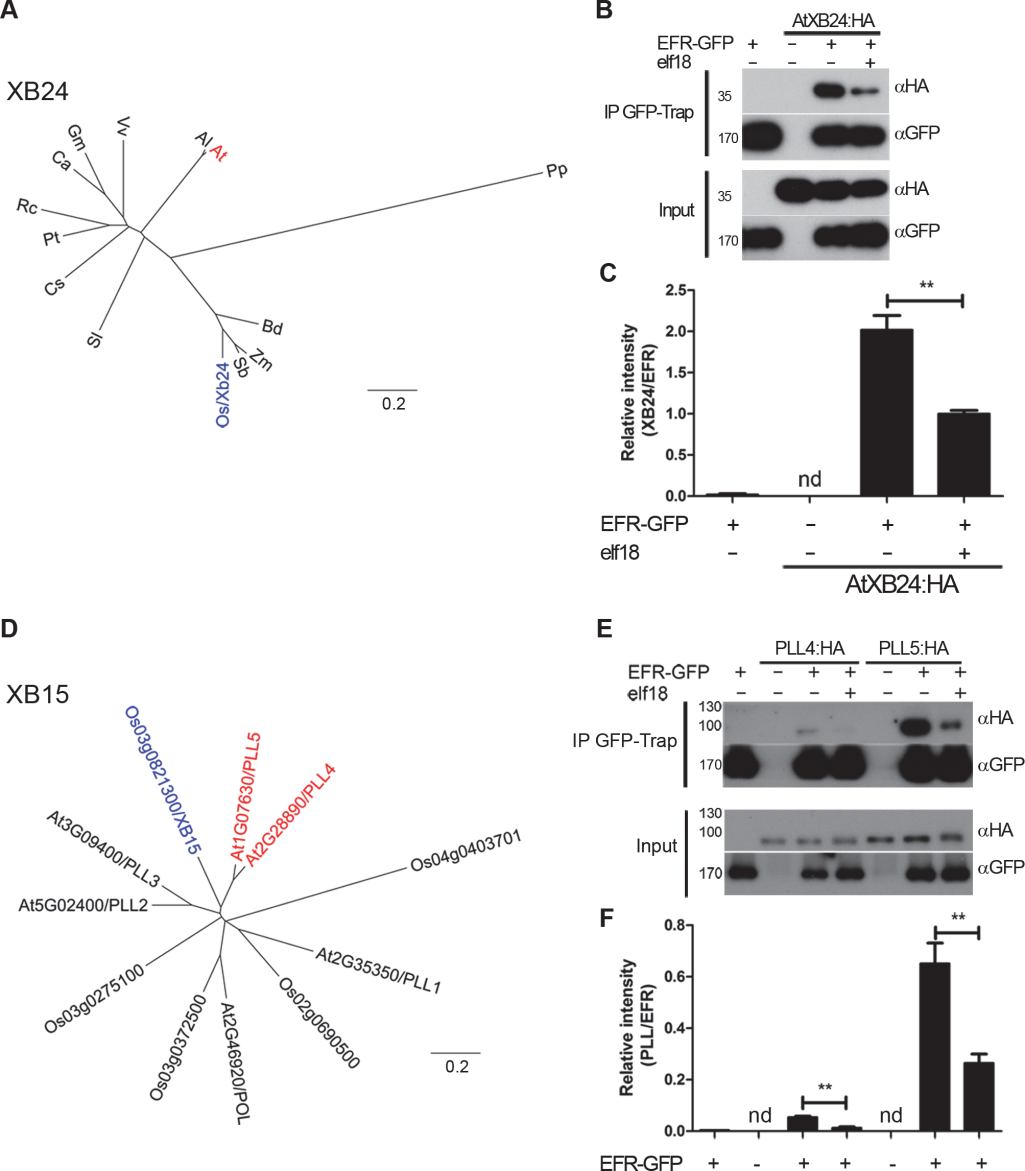
EFR associates dynamically with the *A. thaliana* orthologues of OsXB15 and OsXB24 *in planta*. A,D. Phylogenetic analysis of XB24 and XB15 in rice and Arabidopsis. OsXB24 is a single gene in all plant genomes analyzed. OsXB15 most closely relates to AtPLL4 and AtPLL5. Distance trees are based on protein sequences aligned with MUSCLE (produced with PHYLIP, using PROTDIST and NEIGHBOR). Accession numbers for sequences used can be found in S4 Table and alignments in S1 and S2 Figs. B,E. Co-immunoprecipitation of AtXB24 or AtPLL4 and AtPLL5 (*A. thaliana* orthologues of OsXB15) with EFR. EFR:GFP and HA-tagged AtXB proteins were co-expressed transiently in *N. benthamiana*. Leaves were treated with 100 nM elf18 (+) or mock treated with water (−) for 10 min. Immunoprecipitation (IP) was performed with GFP-Trap agarose beads. Western blots were performed with HRP-conjugated anti-GFP or anti-HA. Experiments were performed at least three times with similar results. C, F. Quantitation of data presented in B and E. Values are the average ratio of AtXB24:HA, PLL4:HA or PLL5:HA to EFR:GFP ±SE (*n* = 3). Asterisks indicate statistical significance of mock compared to elf18 treated, *p* < 0.01 (Student’s t-test). nd is not determined.

XA21 associates with XB24, which possesses ATPase activity and is a negative regulator of XA21 function [50]. A mutation in the ATPase active site of rice XB24 abolished its activity [50]. Phylogenetic analysis of XB24 revealed that orthologues are present as a single copy gene in every plant genome analyzed, including moss, monocots and dicots (Fig. 4A). Interestingly, Arabidopsis XB24 (AtXB24) has a deletion of the ATPase signature present in rice XB24 (S5 Fig.). In addition, rice XB24 appears to be the only XB24 homologue to contain this motif, suggesting that ATPase activity may not be critical for the *in vivo* function of XB24 in some species.

The association between the previously uncharacterized Arabidopsis orthologue AtXB24 (At2g35900) and EFR was assessed. Association of HA-tagged AtXB24 with EFR-GFP was observed after transient co-expression in *N. benthamiana* and co-immunoprecipitation (Fig. 4B,C). Notably, a reduction of the association of AtXB24 was observed following elf18 treatment, indicating a partial dissociation from EFR. This pattern of dissociation is similar to that observed in rice with the interaction between XA21 and XB24, where interaction was only observed in uninfected tissue and XB24 dissociated within 24 hours of *Xoo* infection [50]. This indicates that AtXB24 and rice XB24 are dynamically related in their association kinetics with RKs.

XB15 is a PP2C that dephosphorylates XA21 *in vitro* and negatively regulates XA21-dependent immunity to *Xoo* [51]. XB15 is related to the Arabidopsis POLTERGEIST (POL) family of PP2Cs [51]. Of this family, XB15 is most closely related to POLTERGEIST LIKE 4 (PLL4; AT2G28890) and 5 (PLL5; AT1G07630) (Fig. 4D), with percentage identity of 59% and 58%, respectively (S6 Fig.). PLL2 (AT5G02400) and 3 (AT3G09400) are less closely related and their expression is either not observed or restricted to roots [68]. It was shown previously that XB15, POL and POLTERGEIST LIKE 1 (PLL1) localize to the plasma membrane via myristoylation and palmitoylation [51,69]. PLL4 and PLL5 are almost identical to POL and PLL1 in the region required for membrane association. Therefore, PLL4 and PLL5 were selected as the most probable orthologues of XB15. We tested if PLL4 and PLL5 could associate with EFR by co-immunoprecipitation following transient expression in *N. benthamiana*. HA-tagged PLL4 and PLL5 expressed well, and both PLL proteins precipitated with EFR in the absence of elf18 treatment (Fig. 4E,F). Interestingly, we observed a reduction in the association of both PLL proteins with EFR following 10 min elf18 treatment (Fig. 4E,F). This result indicates that activation of EFR results in dissociation of PLL4 and PLL5 from the complex.

Notably, the kinetics of association between XB15 to XA21 in rice seems different as the complex could only be observed 12–24 hours post-infection with *Xoo* [51]. However, since XA21 protein levels similarly increase during infection [51], it is difficult to determine the relative association of XB15 to XA21. Based on our results, it will be interesting upon identification of the XA21 ligand to test if XA21 activation also leads to XB15 dissociation.

### The Arabidopsis XB15 orthologues PLL4 and PLL5 play a role in EFR-mediated immunity

The dynamic association between EFR and the Arabidopsis XB15 and XB24 orthologues (namely PLL4, PLL5 and AtXB24, respectively) indicated that they play a potential role in EFR-mediated immunity. To test this hypothesis we obtained two independent T-DNA insertion lines for *PLL4, PLL5* and *AtXB24* each with altered transcript levels (S7 Fig.). Morphologically, *Atxb24* lines were indistinguishable from wild-type, however *pll5-1* exhibits darker rosette leaves that are also altered in shape and curling, as previously described [68] (S8 Fig.). The lack of morphological alterations in *pll5-3* is presumably due to the residual expression of *PLL5* in this line (S7 Fig.). We also generated a double *pll4-1 pll5-1* mutant by crossing. The *pll4-1 pll5-1* has a morphology closer to wild-type plants, suggesting some degree of antagonism between *PLL4* and *PLL5* during development as previously described [68].

**Figure 5:**
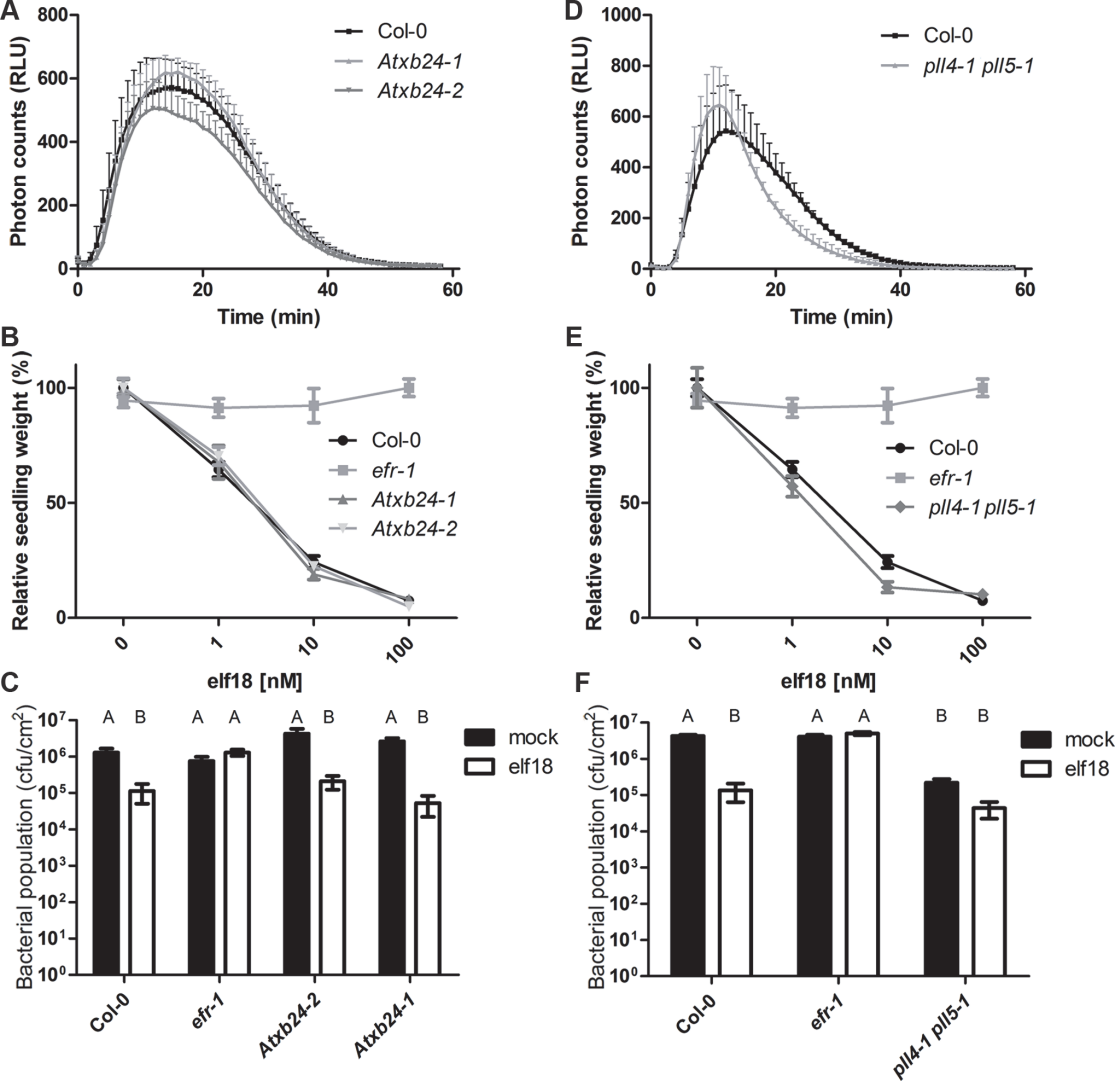
AtXB4 and PLL4/5 play a role in EFR-mediated signaling. A,D. Elf18-induced ROS burst. Leaf discs were treated with 100nM elf18 treatment. Values are averages ±SE (*n* = 3, representing independent biological experiments with 12 samples in each experiment). Plant lines were not statistically different, *p* > 0.05 (two-way ANOVA). The reduced ROS burst in *Atxb24-2* and *pll4-1 pll5-1* lines was observed in 5/6 and 6/7 experiments, respectively. B,E. Seedling growth inhibition in response to increasing dose of elf18. Seedlings were grown in the presence of elf18 and seedling fresh weight recorded 10 days post-treatment. Values are averages ±SE (*n* = 12). Plant lines were not statistically different, *p* > 0.05 (two-way ANOVA). The experiment was repeated three times with similar results. C,F. Elf18-induced resistance to Pto DC3000. Plants were pre-treated with mock or 1 μM elf18 for 24 h before infection with *Pto* DC3000 (OD_600_ = 0.0002). Bacterial populations were scored at 2 dpi. Values are averages ±SE (*n* = 4). Letters indicate statistically significant differences from Tukey’s HSD mean separation (*p* < 0.05). Infections were repeated at least four times with similar results.

To test the involvement of PLL4, PLL5 and AtXB24 in EFR-mediated signaling we determined the extent of the ROS burst, MAPK activation, seedling growth inhibition and induced resistance following treatment with elf18 in these mutants (Figs. 5, S9 and S10). The elf18-induced ROS burst was slightly altered in *Atxb24-2* (Fig. 5A). While this effect was minor and not statistically significant, it was reproducibly observed in 5 out of 6 independent experiments. *Atxb24-1* were wild-type for ROS generation; this is presumably due to the presence of remaining *AtXB24* transcripts in this mutant (S7A,B Fig.). Both *Atxb24-1* and *Atxb24-2* responded similar to wild-type Col-0 plants in the MAPK activation, induced resistance and seedling growth inhibition assays (Figs. 5 B,C, S9 and S10. In rice, reduction in *XB24* transcript levels only improves resistance to *Xoo* in the presence of XA21 and *XB24* overexpression diminishes resistance via destabilization of XA21 following activation [50]. The reduced expression in the knock-down line *Atxb24-2* only slightly reduced elf18-induced ROS burst; while on the basis of XB24 function in rice one may have expected the opposite. In addition, we did not observe a significant reduction in EFR levels upon XB24 co-expression or elf18 treatment (Fig. 4B). Given the lack of ATPase motif in the Arabidopsis homologue (S5 Fig.), it may be that the mechanism of action is different and AtXB24 is acting a positive regulator (at least of the elf18-induced ROS burst) in Arabidopsis, rather than a negative regulator, as it does in rice.

The single *pll* mutants did not show any significant alterations in elf18-induced ROS burst, MAPK activation, seedling growth inhibition or induced resistance (S9–S10 Figs.). The double mutant was also unaltered in elf18-induced MAPK activation (S10 Fig.). However, the double *pll4-1 pll5-1* exhibited slight increase in the amplitude of ROS burst in response to elf18 treatment, which also peaked earlier than in wild-type leaves (Fig. 5D). This effect was reproducibly observed in 6 out of 7 independent experiments, although it is not statistically significant. Elf18-induced seedling growth inhibition was also altered in *pll4-1 pll5-1* seedlings, being ∼10% more sensitive to 1 and 10 nM elf18 than Col-0 seedlings (Fig. 5E). Notably, the *pll4-1 pll5-1* plants exhibited improved resistance to *Pto* already in the absence of elf18 treatment, restricting by ∼1 log bacterial growth compared to wild-type Col-0 (Fig. 5F). Elf18 treatment resulted in a further reduction in bacterial population, although this was to a lesser degree than in wild-type (Fig. 5F). These results revealed that PLL4 and PLL5 act as negative regulators of immunity in Arabidopsis, similarly as XB15 in rice.

In rice, a transposon-insertional *XB15* mutant exhibits constitutive cell death lesions, enhanced XA21-mediated resistance and induction of defense gene expression. We did not observe cell death or constitutive *PR1* or MAPK activation in *pll4-1 pll5-1* plants under our conditions (S10 Fig.), suggesting that there may be a greater level of redundancy in this regulation system in Arabidopsis than in rice, or that the outputs of the PRRs, which they control, do not induce cell death. PLL4 and PLL5 may negatively regulate several PRRs in addition to EFR, such that in *pll4-1 pll5-1* the observed increased resistance may be a result of the cumulative hyper-activation of multiple PRRs by *Pto*. Such promiscuity could account for the morphological phenotypes observed in *pll5-1*, as PLL5 may also associate with several RKs involved in development, as previously suggested for POL and PLL1 [70]. However, these morphological alterations most likely do not account for the immune defects observed, since *pll5-1* is more severely altered in development but does not display any immunity-related phenotype (S8–S10 Figs.).

### Conclusion

The transfer of PRRs between plant species is an approach by which durable broad-spectrum disease resistance could be achieved [17,18]. Thus, there is a great interest in understanding the mechanisms of PRR-mediated resistance and how these could be applied biotechnologically. The recent successful transfer of PRRs across plant families [21,23–25,71] indicates that signaling components acting downstream of PRRs are conserved, and thus that transferred PRRs can simply ‘plug-in’ into these pathways somehow as ‘antennae’ being newly connected to a pre-existing communication network. We have here used two related PRRs from the same sub-family of LRR-RKs (XII), namely Arabidopsis EFR and rice XA21, to test directly the extent of conservation in PRR-mediated signaling between two different plant clades. Although monocots and dicots diverged over 150 million years ago [72], we revealed here that the XA21 intracellular domain is functional at activating immune signaling in Arabidopsis in response to the PAMP elf18 when fused to the extracellular domain of EFR, indicating that the XA21 intracellular domain is able to interact biochemically with similar components as EFR in Arabidopsis. Indeed, we demonstrated that the EFR:XA21 chimera associates with the co-receptor BAK1 and the cytoplasmic kinase BIK1, which are both common interactors and regulators of multiple PRRs in Arabidopsis. Notably, the association of the chimera with BAK1 could only be observed upon elf18 treatment, while BIK1 dissociated from the chimera after elf18 perception. These ligand-dependent dynamic associations clearly indicate the functional biochemical interactions between the EFR:XA21 chimera and these key Arabidopsis immune components.

Motivated by the ability of the XA21 intracellular domain to channel immune signaling in Arabidopsis, we hypothesized that EFR-mediated signaling may employ signaling components that are orthologous to those used by XA21 in rice. Indeed, we showed that the putative ATPase AtXB24 and the PP2Cs PLL4 and PLL5 associate with EFR and that elf18 perception leads to the partial dissociation of these complexes. AtXB24 appeared to be a positive regulator of elf18-triggered ROS burst in Arabidopsis, but did not affect other responses triggered by elf18, such as seedling growth inhibition and induced resistance to *Pto* DC3000. In contrast, PLL4 and PLL5 are partially redundant in negatively regulating elf18-induced ROS burst and seedling growth inhibition, as well as immunity to *Pto* DC3000. It will be interesting in the future to decipher the mechanisms by which these proteins regulate EFR.

Notably, in parallel to this study, we recently demonstrated that EFR is functional in rice, confers slight enhanced resistance to hypovirulent bacterial strains, and recruits the same signaling components as XA21 (Schwessinger et al., submitted). Together, these studies provide experimental evidence for the overall conservation of immune signaling between monocots and dicots, which paves the way for further PRR transfers between these clades. Our work also illustrates the usefulness of comparative studies between monocots and dicots, as well as between plant models and crops, to reveal novel immune components.

## Materials and Methods

### Plant material and growth conditions

*Arabidopsis thaliana* was grown in soil under short day condition (10 h light) at 20–22°C. *Nicotiana benthamiana* plants were grown in soil at 22°C with a photoperiod of 16 h. T-DNA insertion lines were obtained from NASC and were genotyped using the primers described in S2 Table.

### Chemicals

Elf18 peptide was obtained from Peptron Inc. (Korea).

### Vector construction

Full length EFR:GFP was cloned by fusing GFP to a cDNA clone of *EFR*, using overlap extension PCR of amplified DNA fragments 1 and 2 (S1 Table). EFR:GFP was cloned into pENTR/D-TOPO and transferred into pEARLEYGATE100 by Gateway cloning. XA21:GFP was cloned as a *Nar*I/*Nhe*I PCR fragment, generated from pUBI:XA21:GFP plasmid [73], into pBIN19g which had been digested with *Cla*I and *Xba*I. Chimeric receptor constructs were produced by overlap extension PCR using respective PCR-amplified DNA fragments (S1 Table). Chimeric PCR products were cloned by Gateway cloning into pEARLEYGATE100, for EFR:XA21:GFP or pEARLEYGATE103, for XA21:EFR:GFP. Cytoplasmic deletion constructs were produced by PCR from EFR or EFR:XA21 cDNA and cloning into pENTR/D-TOPO then subcloning into pEARLEYGATE103 by Gateway cloning.

35S promoter HA-tagged *PLL4, PLL5* and *AtXB24* constructs were amplified by PCR from *Arabidopsis* leaf cDNA and cloned using Gateway cloning into pGWB14. All constructs were sequenced to ensure they were correct and no mutations had been introduced by PCR. PCR primers used for cloning and generation of chimeras can be found in S1 Table.

### Agrobacterium-mediated transient expression

Transient expression was performed by diluting an overnight culture *A. tumefaciens* GV3101 containing the appropriate expression plasmid to OD_600_ = 0.2 in 10mM MES (pH5.6), 10mM MgCl_2_ and 100μM acetosyringone. Four-week-old *N. benthamiana* leaves were syringe infiltrated. Samples were collected for analysis two days later.

### ROS burst

Oxidative burst measurements were performed as described previously [39]. Leaf discs were taken from 4-week-old Arabidopsis plants or *N. benthamiana* 2 days after transient infection with Agrobacterium.

### Seedling growth inhibition

Growth inhibition measurements were performed as described previously [39].

### MAPK activation

Leaf discs were incubated overnight in water then treated with 100nM elf18 and samples were frozen in liquid nitrogen. Samples to test for a potential constitutive activation of MAPKs were frozen directly from the plant. Proteins were extracted by grinding samples and boiling for 5 min in Laemmli sample buffer. Protein extracts were run on 10% SDS PAGE gel and transferred onto PVDF membrane. Western blots were performed with anti-p44/42 MAPK (Cell Signaling Technology) and HRP conjugated anti-rabbit antibodies (Sigma).

### Quantitative RT-PCR

Quantitative RT-PCR was performed as described previously [74], with the exception that adult leaves of soil-grown plants were used.

### Bacterial infections

Induced resistance was performed by syringe infiltrating 1μM elf18, or water for mock treatments, into 4-week-old Arabidopsis leaves. The following day pretreated leaves were syringe infiltrated with *Pto* DC3000 suspended in 10mM MgCl_2_ at an OD_600_ of 0.0002. Samples were obtained 2 days post infection to determine bacterial population. Spray inoculations were performed by spraying *Pto* DC3000 *COR^−^* suspended in 10mM MgCl_2_ and 0.02% Silwet L-77 at an OD_600_ = 0.2. Plants were covered and samples were collected 3 days post infection to determine bacterial populations.

Agrobacterium infections of Arabidopsis plants were performed as described previously [13]. Samples for colorimetric GUS staining and quantitative MUG assays were taken 3 days post infection.

### Immunoprecipitation

Leaf tissue was treated with 100nM elf18 by vacuum infiltration for 10min, then frozen in liquid nitrogen. Protein was extracted from *N. benthamiana* or Arabidopsis in 5 ml per g of tissue of extraction buffer (150 mM Tris-HCl pH 7.5; 150 mM NaCl; 10% glycerol; 1% IGEPAL CA630; 10mM DTT; protease inhibitor cocktail (Sigma)). For *N. benthamiana* 1% PVPP was added. Extracts were centrifuged at 10000g for 15 min and filtered through Miracloth. GFP-Trap agarose beads (manufacturer) were added and extracts were incubated for 3 hr at 4°C. Beads were washed with extraction buffer without DTT and containing 0.1% IGEPAL CA630. Protein was eluted with SDS loading buffer by boiling for 4 min. Western blotting was performed with HRP conjugated anti-GFP (Santa Cruz) or anti-HA (Roche) or unconjugated anti-BAK1 described by Schwessinger *et al*. [56].

### Phylogenetic analysis

Protein sequences were obtained from NCBI and aligned using MUSCLE[75]. Accession numbers of these sequences can be found in S4 Table and alignments used for phylogenetic analysis are shown in S1 and S2 Figs. Phylogenetic analysis was performed using PHYLIP [76]. Trees were constructed using PRODIST and NEIGHBOR.

### Statistical analysis

Graphpad prism was used to conduct statistical analysis. One-way analysis of variance was performed with post-hoc Turkey’s HSD comparison. Alternatively Student’s t-tests were performed for paired comparisons.

## Supporting Information

S1 Fig Localization of EFR, XA21 and chimeric receptors. Confocal microscopy of GFP tagged EFR (A), XA21 (B), EFR:XA21 (C) and XA21:EFR (D) in *N. benthamiana*. Arrows indicate the perinuclear endoplasmic reticulum. All images are taken at the same scale.
(TIF)

S2 Fig Marker gene expression in EFR and EFR:XA21 transgenic Arabidopsis. Quantitative RT-PCR was used to determine *PR1* (A), *FRK1* (B), *PHI1* (C) and *NHL10* (D) in response to elf18 treatment. Treatments were performed with 100 nM elf18, except for *PR1* expression where 1 μM was used. Expression is relative to *U-box* expression and was calculated by the comparative CT method. Values are averages ±SE (*n* = 6). Asterisks indicate significantly different values as compared to *efr-1* at *p* <0.05 (one-way ANOVA).
(TIF)

S3 Fig Expression of EFR, XA21, EFR:XA21 or XA21:EFR does not lead to constitutive MAPK activation or *PR1* expression. A. MAPK activity of untreated transgenic lines. MAPK activation in Col-0 by 15 min flg22 treatment (flg22) is shown as a positive control. Western blots were performed with anti-p44/42 MAPK antibodies. Experiments were performed three times with similar results. B. *PR1* expression in untreated transgenic lines. Expression is relative to U-box expression and was calculated by the comparative CT method. Values are averages ±SE (*n* = 6). No significant difference was seen between lines, *p* < 0.05, as determined by one-way ANOVA.
(TIF)

S4 Fig BAK1 interaction is dependent on the extracellular domain. A. Co-immunoprecipitation of transiently-expressed EFRΔcyt:GFP and EFR:XA21Δcyt::GFP with BAK1:HA in *N. benthamiana*. Leaves were treated with 100 nM elf18 (+) or mock treated with water (−) for 10 min. Immunoprecipitation (IP) was performed with GFP-Trap agarose beads. Western blots were performed with HRP-conjugated anti-GFP and anti-HA antibodies. B. Elf18-induced ROS production in *N. benthamiana* expressing EFR:GFP, EFR:XA21:GFP, EFRΔcyt:GFP and EFR:XA21Δcyt:GFP. ROS production is represented as the total of photons emitted during 40 min after treatment with 100 nM elf18. Values are averages ±SE (*n* = 12). Asterisks indicate statistical difference from *GUS* transformed leaves, *p* < 0.0001 (Student’s t-test). Experiments were performed at least twice with similar results.
(TIF)

S5 Fig Multiple alignment of XB24 orthologues. Alignment of XB24 homologues used to create the phylogenetic tree in Fig. 4A. The ATPase motif of XB24 is highlighted in red. Accession numbers are listed in S4 Table.
(DOCX)

S6 Fig Multiple alignment of XB15 orthologues. Alignment of the XB15/POL homologues used to create the phylogenetic tree in Fig. 4D. Accession numbers are listed in S4 Table.
(DOCX)

S7 Fig Description of *Atxb24, pll4* and *pll5* T-DNA mutants. A,C,E. Schematic representation of the T-DNA insertion sites in *AtXB24, PLL4* and *PLL5*. Arrows above the genes indicate the positions of primers used for RT-PCR. B,D,F. Semi-quantitative RT-PCR of *Atxb24, pll4* and *pll5* T-DNA insertion lines. Primers are listed in S3 Table.
(TIF)

S8 Fig Phenotype of Col-0, *pll4, pll5, pll4 pll5*, and *Atxb24* adult plants. Plants were photographed 6 weeks after sowing.
(TIF)

S9 Fig *pll4* and *pll5* single mutants phenotypes. A. Elf18-induced ROS burst. Leaf discs were treated with 100nM elf18 treatment. Values are averages ±SE (*n* = 3, representing independent biological experiments with 12 samples in each experiment). Plant lines were not statistically different, *p* > 0.05 (two-way ANOVA). B. Seedling growth inhibition in response to increasing dose of elf18. Seedlings were grown in the presence of elf18 and seedling fresh weight recorded 10 days post-treatment. Values are averages ±SE (*n* = 12). Plant lines were not statistically different, *p* > 0.05 (two-way ANOVA). The experiment was repeated three times with similar results. C. Elf18-induced resistance to Pto DC3000. Plants were pre-treated with mock or 1 μM elf18 for 24 h before infection with *Pto* DC3000 (OD_600_ = 0.0002). Bacterial populations were scored at 2 dpi. Values are averages ±SE (*n* = 4). Letters indicate statistically significant differences from Tukey’s HSD mean separation (*p* < 0.05). Infections were repeated at least four times with similar results.
(TIF)

S10 Fig MAPK activation and basal *PR1* expression is not altered in *pll4, pll5* or *Atxb24* mutants. A. MAPK activation in *pll4, pll5, pll5-1 pll4-1* and *Atxb24* insertion lines after elf18 treatment. Western blots were performed simultaneously and exposed for the same time. Western blots were performed with anti-p44/42 MAPK antibodies. Even loading is demonstrated by Coomassie brilliant blue (CBB) staining. The experiment was repeated twice with similar results. B. Basal *PR1* expression in *pll4, pll5* and *Atxb24* lines. Expression is relative to U-box expression and was calculated by the comparative CT method. Values are averages ±SE (*n* = 6). No significant difference was seen between lines, *p* < 0.05, as determined by one-way ANOVA.
(TIF)

S1 Table Primers used for cloning. (XLSX)

S2 Table Primers used for genotyping. (XLSX)

S3 Table Primers used for RT-PCR. (XLSX)

S4 Table Sequences used for phylogenetic analyses. (XLSX)
