# Supplementary figures and images for: The Phylogenetically-Related Pattern Recognition Receptors EFR and XA21 Recruit Similar Immune Signaling Components in Monocots and Dicots
Source: PLoS Pathog. 2015 Jan 21;11(1):e1004602. doi: 10.1371/journal.ppat.1004602 (PMC4301810; doi:10.1371/journal.ppat.1004602)

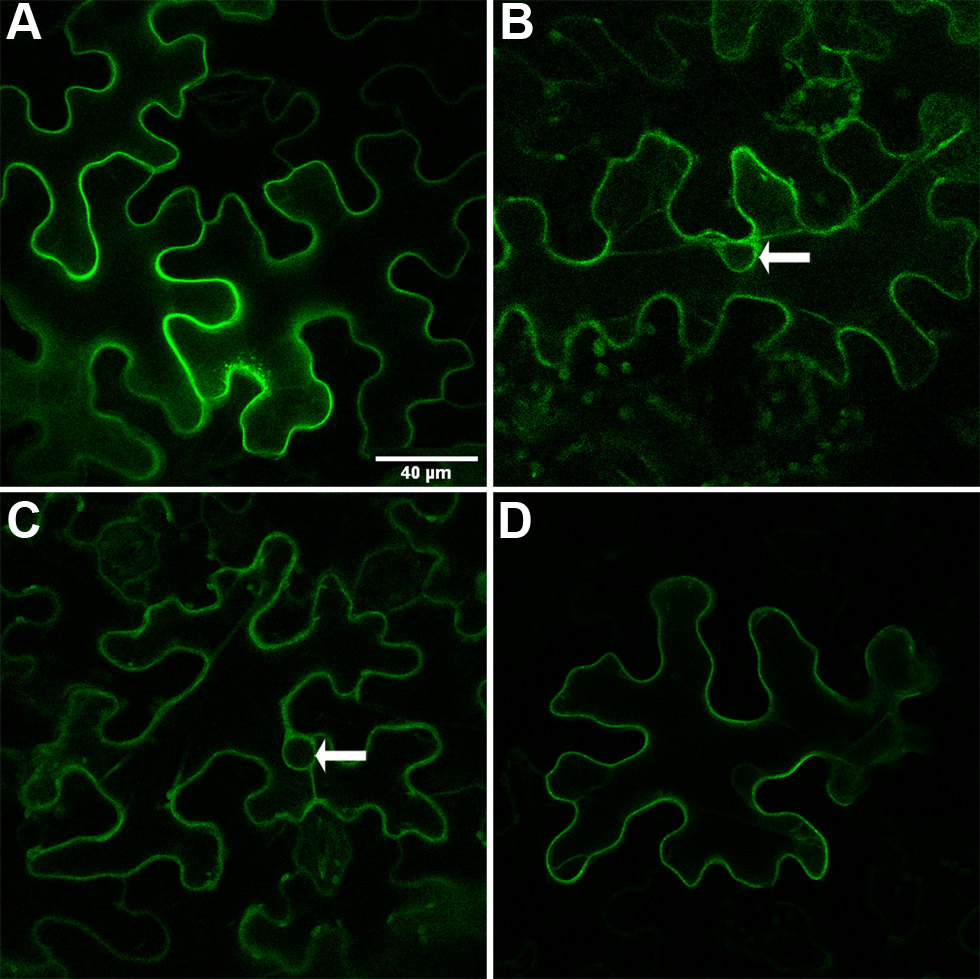

Supplement: S1 Fig — Confocal microscopy of GFP tagged EFR (A), XA21 (B), EFR:XA21 (C) and XA21:EFR (D) in N. benthamiana. Arrows indicate the perinuclear endoplasmic reticulum. All images are taken at the same scale. (TIF) [file ppat.1004602.s001.tif]

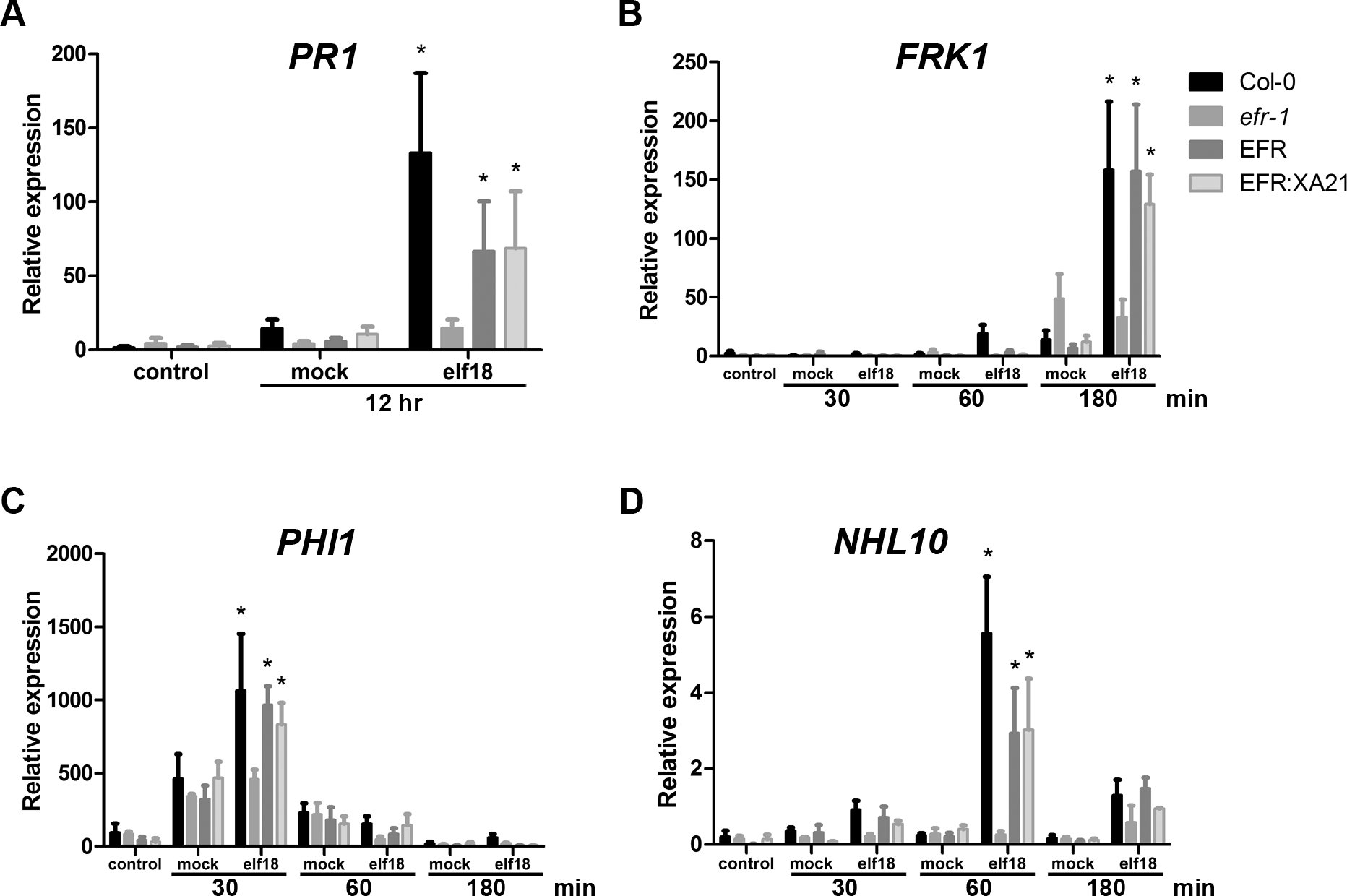

Supplement: S2 Fig — Quantitative RT-PCR was used to determine PR1 (A), FRK1 (B), PHI1 (C) and NHL10 (D) in response to elf18 treatment. Treatments were performed with 100 nM elf18, except for PR1 expression where 1 μM was used. Expression is relative to U-box expression and was calculated by the comparative CT method. Values are averages ±SE (n = 6). Asterisks indicate significantly different values as compared to efr-1 at p <0.05 (one-way ANOVA). (TIF) [file ppat.1004602.s002.tif]

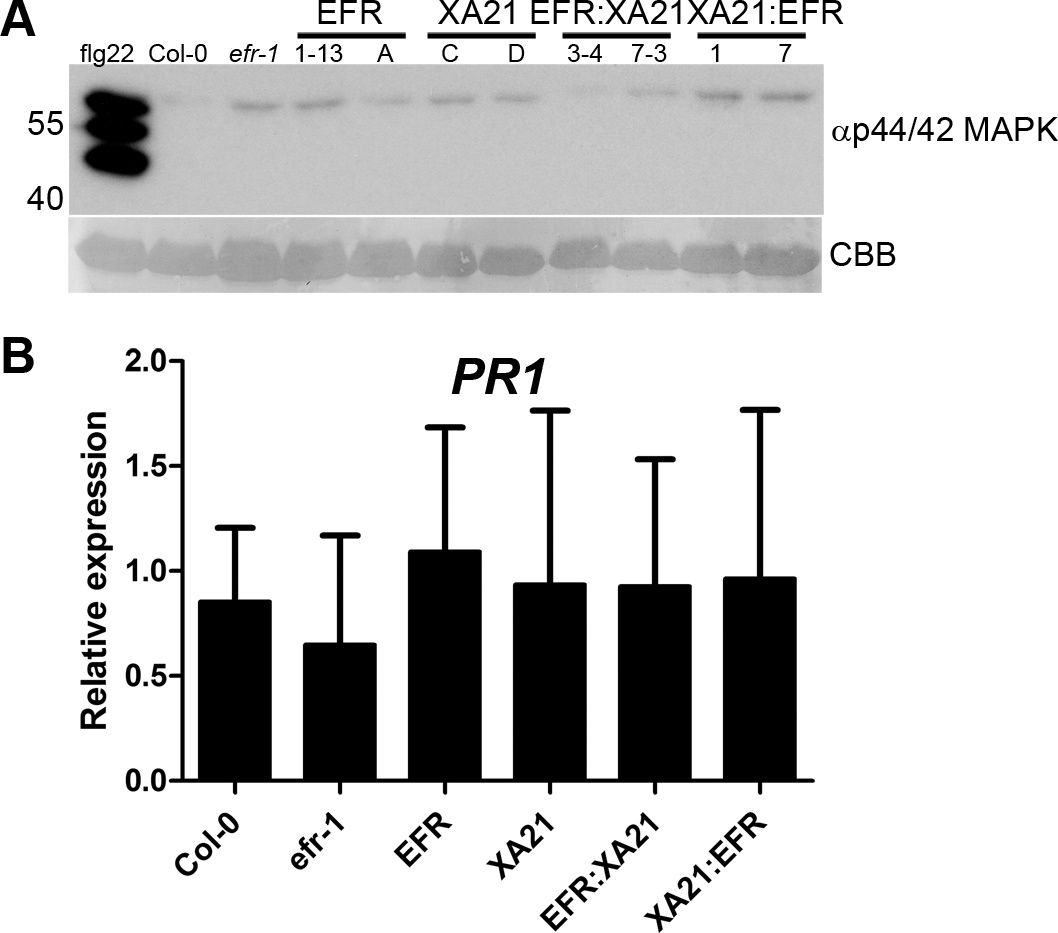

Supplement: S3 Fig — A. MAPK activity of untreated transgenic lines. MAPK activation in Col-0 by 15 min flg22 treatment (flg22) is shown as a positive control. Western blots were performed with anti-p44/42 MAPK antibodies. Experiments were performed three times with similar results. B. PR1 expression in untreated transgenic lines. Expression is relative to U-box expression and was calculated by the comparative CT method. Values are averages ±SE (n = 6). No significant difference was seen between lines, p < 0.05, as determined by one-way ANOVA. (TIF) [file ppat.1004602.s003.tif]

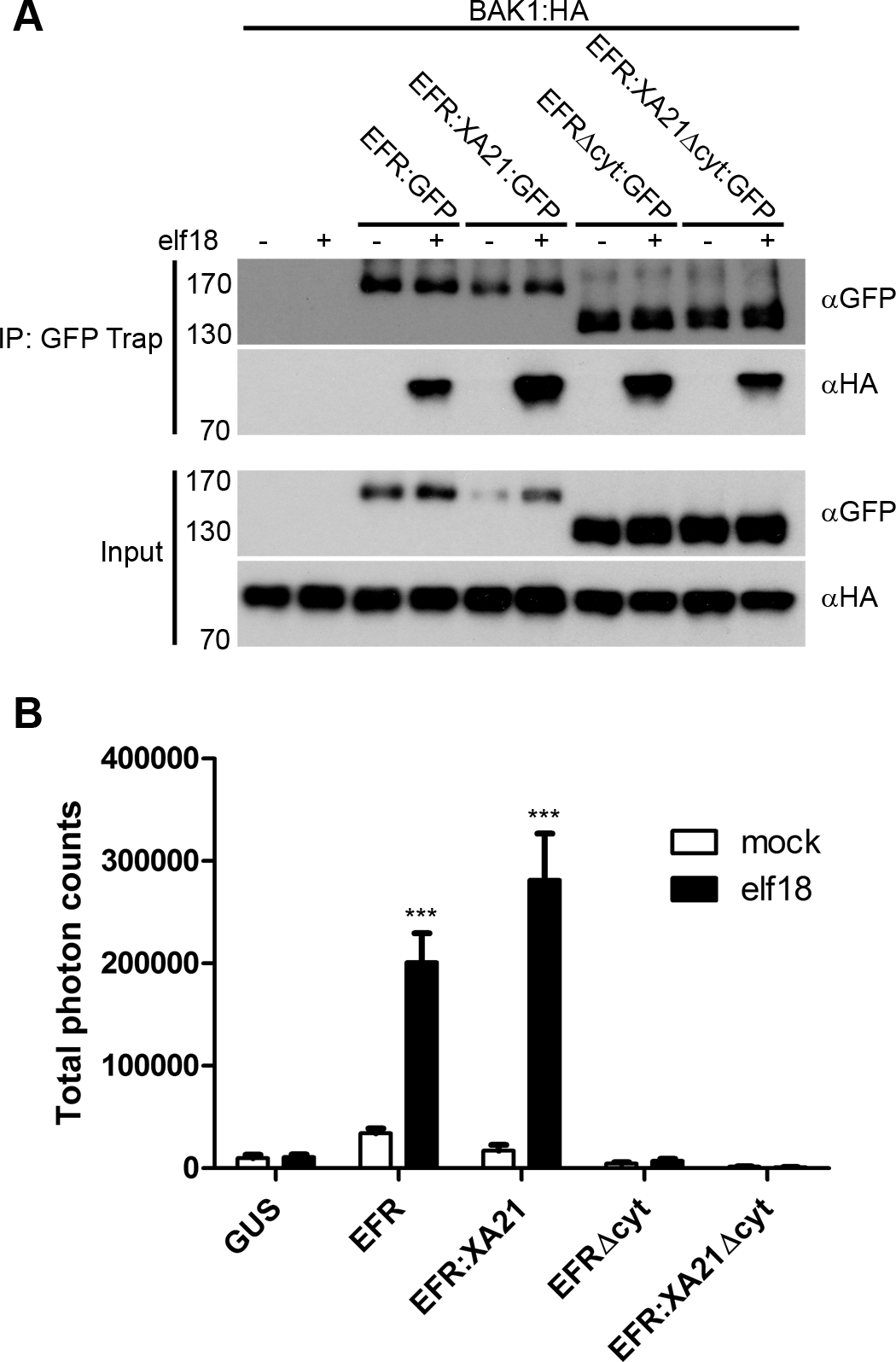

Supplement: S4 Fig — A. Co-immunoprecipitation of transiently-expressed EFRΔcyt:GFP and EFR:XA21Δcyt::GFP with BAK1:HA in N. benthamiana. Leaves were treated with 100 nM elf18 (+) or mock treated with water (−) for 10 min. Immunoprecipitation (IP) was performed with GFP-Trap agarose beads. Western blots were performed with HRP-conjugated anti-GFP and anti-HA antibodies. B. Elf18-induced ROS production in N. benthamiana expressing EFR:GFP, EFR:XA21:GFP, EFRΔcyt:GFP and EFR:XA21Δcyt:GFP. ROS production is represented as the total of photons emitted during 40 min after treatment with 100 nM elf18. Values are averages ±SE (n = 12). Asterisks indicate statistical difference from GUS transformed leaves, p < 0.0001 (Student’s t-test). Experiments were performed at least twice with similar results. (TIF) [file ppat.1004602.s004.tif]

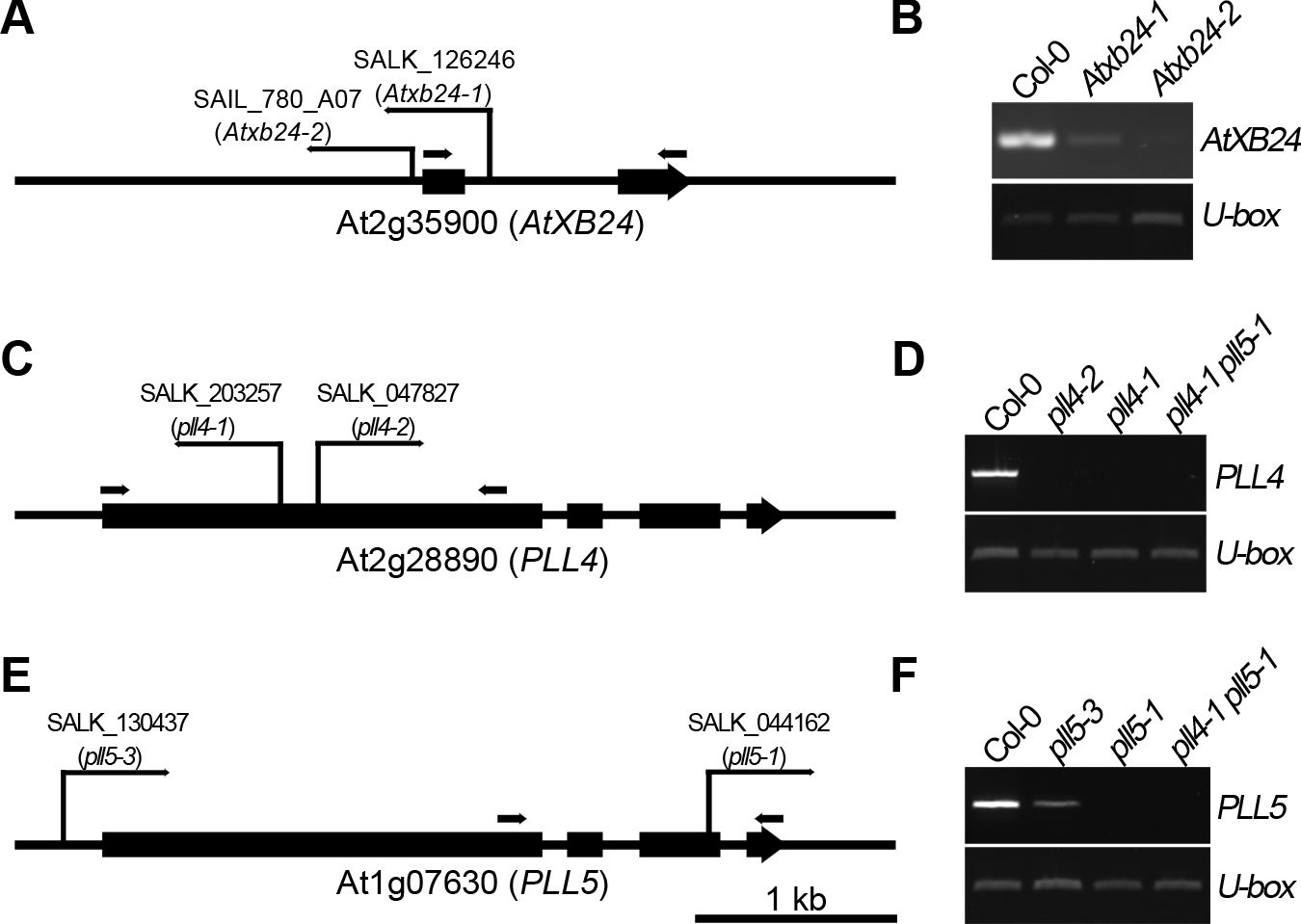

Supplement: S7 Fig — A,C,E. Schematic representation of the T-DNA insertion sites in AtXB24, PLL4 and PLL5. Arrows above the genes indicate the positions of primers used for RT-PCR. B,D,F. Semi-quantitative RT-PCR of Atxb24, pll4 and pll5 T-DNA insertion lines. Primers are listed in S3 Table. (TIF) [file ppat.1004602.s007.tif]

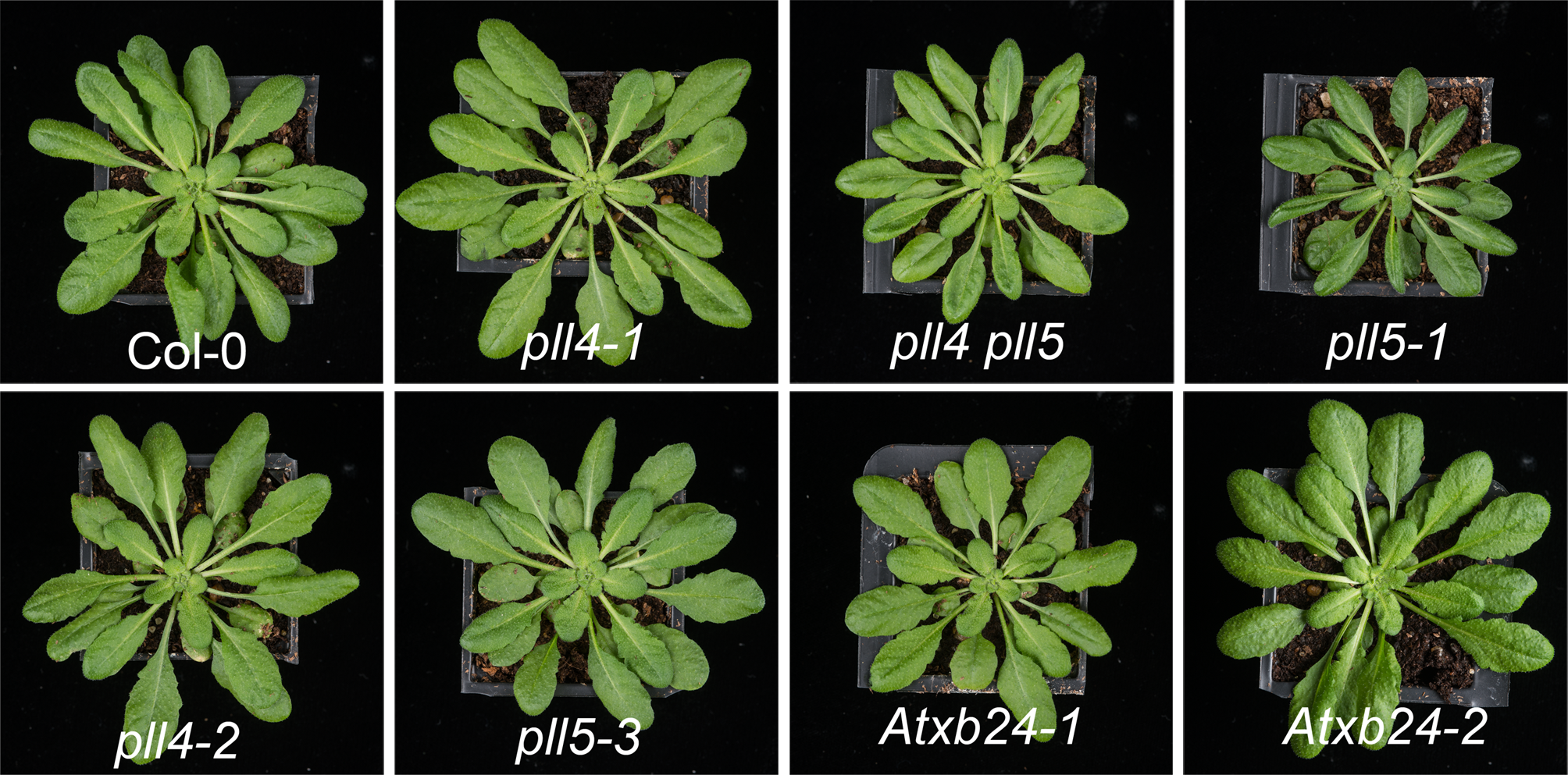

Supplement: S8 Fig — Plants were photographed 6 weeks after sowing. (TIF) [file ppat.1004602.s008.tif]

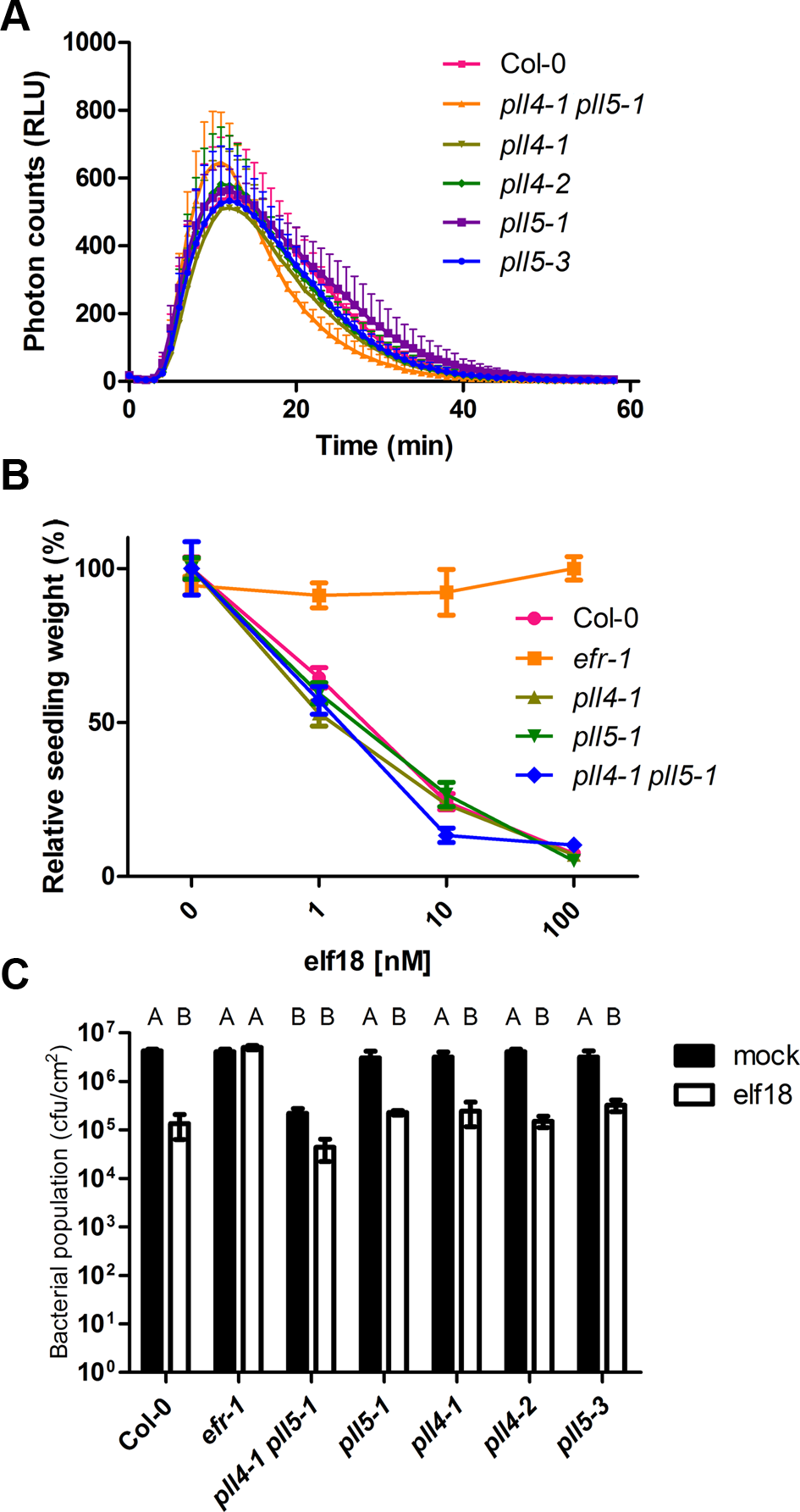

Supplement: S9 Fig — A. Elf18-induced ROS burst. Leaf discs were treated with 100nM elf18 treatment. Values are averages ±SE (n = 3, representing independent biological experiments with 12 samples in each experiment). Plant lines were not statistically different, p > 0.05 (two-way ANOVA). B. Seedling growth inhibition in response to increasing dose of elf18. Seedlings were grown in the presence of elf18 and seedling fresh weight recorded 10 days post-treatment. Values are averages ±SE (n = 12). Plant lines were not statistically different, p > 0.05 (two-way ANOVA). The experiment was repeated three times with similar results. C. Elf18-induced resistance to Pto DC3000. Plants were pre-treated with mock or 1 μM elf18 for 24 h before infection with Pto DC3000 (OD600 = 0.0002). Bacterial populations were scored at 2 dpi. Values are averages ±SE (n = 4). Letters indicate statistically significant differences from Tukey’s HSD mean separation (p < 0.05). Infections were repeated at least four times with similar results. (TIF) [file ppat.1004602.s009.tif]

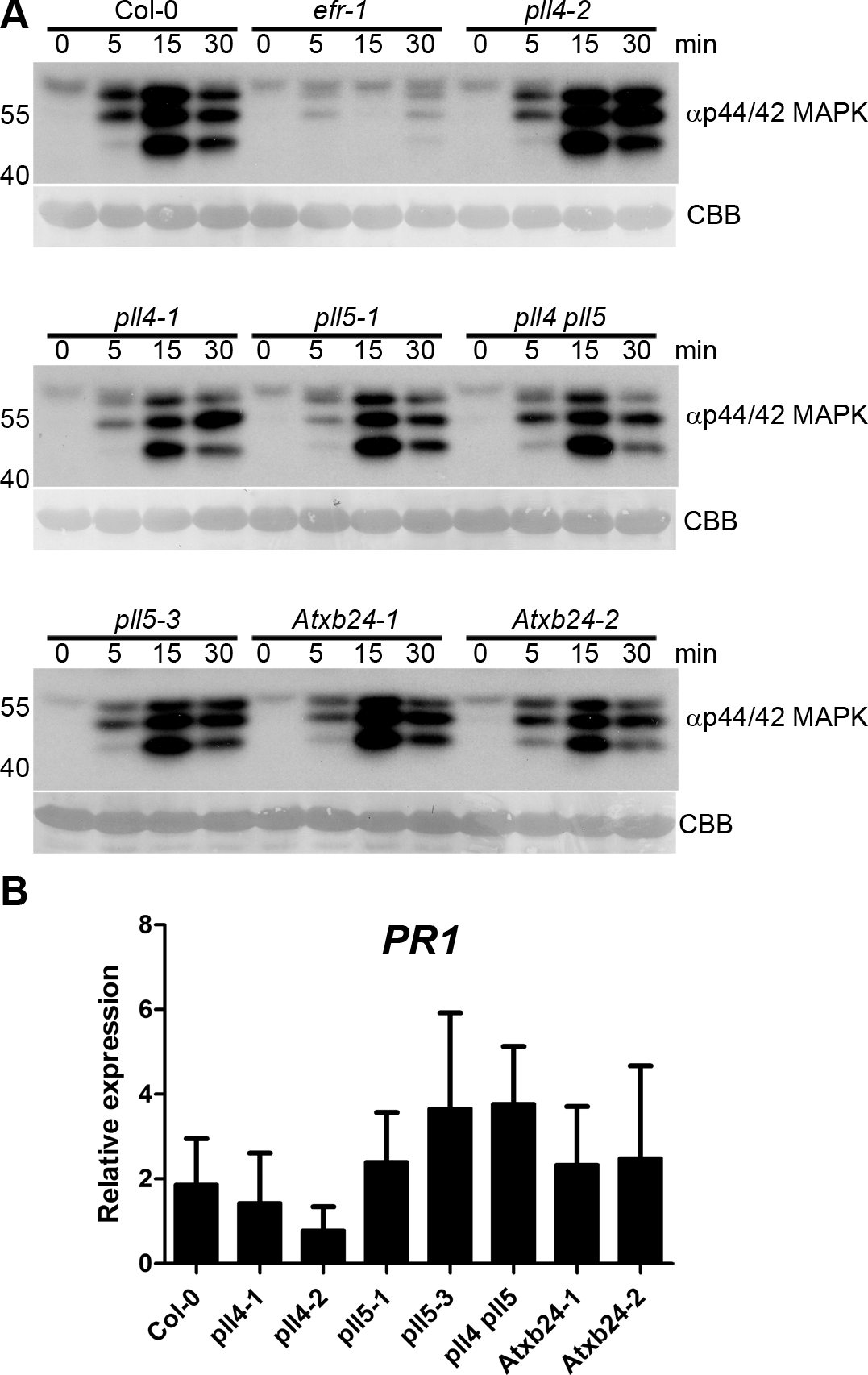

Supplement: S10 Fig — A. MAPK activation in pll4, pll5, pll5-1 pll4-1 and Atxb24 insertion lines after elf18 treatment. Western blots were performed simultaneously and exposed for the same time. Western blots were performed with anti-p44/42 MAPK antibodies. Even loading is demonstrated by Coomassie brilliant blue (CBB) staining. The experiment was repeated twice with similar results. B. Basal PR1 expression in pll4, pll5 and Atxb24 lines. Expression is relative to U-box expression and was calculated by the comparative CT method. Values are averages ±SE (n = 6). No significant difference was seen between lines, p < 0.05, as determined by one-way ANOVA. (TIF) [file ppat.1004602.s010.tif]
