# Supplementary material for: The Phylogenetically-Related Pattern Recognition Receptors EFR and XA21 Recruit Similar Immune Signaling Components in Monocots and Dicots
Source: PLoS Pathog. 2015 Jan 21;11(1):e1004602. doi: 10.1371/journal.ppat.1004602 (PMC4301810; doi:10.1371/journal.ppat.1004602)
Supplement: S5 Fig — Alignment of XB24 homologues used to create the phylogenetic tree in Fig. 4A. The ATPase motif of XB24 is highlighted in red. Accession numbers are listed in S4 Table. (DOCX) [file ppat.1004602.s005.docx]

Zm MGWRWHDDD-----------GDSGR------GLGGVPDLAG------------------G 25

Sb MGWRWHDDD----------DGDSGR------GLGGVPDLAG------------------G 26

Os/Xb24 MGWRWHDDG-----------DDGGR------GLGDIPDLAGGG----------------G 27

Bd MGWRWHDEEG---------EGDGDR------GLGDIPG---------------------R 24

At MGWIWIDDS------------SASKA-----GADLTDKPLG-D----------------S 26

Al MGWVWIDDS------------SASKA-----GTDLTDKPLG-D----------------S 26

Cs MGWVWRDDT------------DDSDS-----FAADADKFNNST----------------S 27

Pt MGWVWRDD----DESDD--------------SFQKNPNYS-------------------S 23

Rc MGWVWRDD----DEKEEFSSSSIGGDISEYKNKNNNPNYS-------------------- 36

Ca MGWVWSDDD-----------------------SQLNT----------------------S 15

Gm MGWVWKDDN-----------------------SDDVR----------------------R 15

Vv MGWVWEDD-------------AGEAN-----TAGDVTGFQN------------P-----N 25

Sl MGWVWINDE---------PKHSGADDFSAVKDFGNPRSSSG-S----------------D 34

Pp MDVRLSRMS----------DGSEGDSRE-GFGNGESIGFSR------------------K 31

*.

Zm GGDGAQCATRRVVQSRCHTEEVE-PGRFVRKCEKTEQLLRDCVGRPSELVESKTENTEED 84

Sb GGEGAQCATRRVVQSRCHTEEVE-PGRFVRKCEKTEQLLRDCVGRPSELVESKTENTEED 85

Os/Xb24 GGDGERCATRRVVQSRCHTEEVE-PGRFVRKCEKTEQLLRDCVGRPSELVESKTENTEED 86

Bd GGEGAHLGTRRVVQSRCRTEEVE-PGRFVRKCEKTEQLLRDCIGRPSELVESKTENTEED 83

At AAAAENCSTTTVVRSQCKTEEVE-PGKFVRKCDKTEEILRHCFGKPSEVVQSNTEHTEED 85

Al SAAAGNCSTTTVVRSQCKTEEVE-PGKFVRKCDKTEEILRHCFGKPSEVVQSNTEHTEED 85

Cs PSSSDWCSTRNVVRSHCKTEEVE-PGKFVRKCERTEEILRDCIGRPTEVIKSNKEITEED 86

Pt SSSGEVCSTRTVVRSQCKTEEVE-PGKFVRKCEKTEEVLRDCLGKPVEVLKSNKEYTEDD 82

Rc SPSDKVCSTRKVVKSQCKTEEVE-PGKFVRKCEKSEEILRECLGEPVEVLKSNKEYTEDD 95

Ca SGSDERYSTRKIVKSQCRTEEVE-PGKFVKKCEKTEELLRTCTGKPVEVLQSNKEYTEED 74

Gm DNSSERCATTKVVKSQCRTEEVE-TGKFVRKCEKTEEILRNCIGKPAEVLQSNKEYTEED 74

Vv LRSEDRCSTRRIVKSQCRTEEVK-PGKFVRKCQKTQQILKDCVGRATEVVQSNEEYTEDD 84

Sl GDGGERCATRKVVSTRCRTEETE-PGKFIRKCEKAEQTFKDCIGRPSEIVESNKEYSEED 93

Pp FDPNDPCTYSTIRKYKCFLEGNDSSGNAVQKCERTEQLLRRCPGRPVEVVKSETEYTEGD 91

: :* * . .*. ::**::::: :: * *.. *:::*: * :* *

Zm VTEEMKSG--SLSLGFPRN-----EPFAFPGLRSDMEALEKDFF---------------- 121

Sb VTEEMKSG--SLSLGFPRN-----EPFAFPGLRSDMEALEKDFF---------------- 122

Os/Xb24 VTDEMKSG--SLSLGFPTN-----EPFAFPGLRSDIEALEKGLF---------------- 123

Bd VTDEMTGA--SHSLGFPAK-----EPFAFPGLRSDIEAIEKGFS---------------- 120

At VTDQMVGR-SALPNQFEDK-----NPLNFPGLRRDVDAIERHFL---------------- 123

Al VTDQMVGR-SALPNQFEER-----NPLNFPGLRRDVDAIERHFL---------------- 123

Cs VTDQMVNRSFSLGSSPSEH-----RPLDFPGLRSDIDAIEQSLF---------------- 125

Pt VTEQVVRG--LLHPGKFED-----VPFDFHGLRGDIQDIERHFL---------------- 119

Rc VTDLVAKG--SSTLGKFDN-----SPFDFPGLRSDIEGLERHFL---------------- 132

Ca ITDEVLRG-RSTTFGSSNSSSDH-GVFDFPGLRSDIEVMERNLF---------------- 116

Gm ITDEVLKG-RSVPFSSSDGAS---GVVDFPGLQNDIEVMERNLL---------------- 114

Vv VTDLVVKG--SAPLGLPEQ-----GHFDFPGLRSDIEAIERNLF---------------- 121

Sl VTDQMTND--SHSIESSS------VPFDFPGLRSDIENIERSFF---------------- 129

Pp AA----TGSTNFWIEGSDR------SLPLPGLHSDVEQDPRSILPRPFTESDWPRSLTQR 141

: . : **: *:: : :

Zm ----------GSLGNV----------LDEAERMANSFVRSFG-FPPAHDSGSSPFRRQ-- 158

Sb ----------GSLGNV----------LDEAERMTNSFIKSFG-FPPVHDRESSPFRRQ-- 159

Os/Xb24 ----------GSIGSF----------LDDAERMTNDFLKSFG-VPSINERESSSFDGQPT 162

Bd ----------GSIGSF----------MEEAERITNDFFKSFG-FPSIDDGEPRRLPRQ-- 157

At ----------SGMKSF----------FDAAEEMTSSLFDIMG---------DHHPSTRRG 154

Al ----------SGMKSF----------FDAAEEMTSSLFDVMG---------DHHPSTRRG 154

Cs ----------GSMKGF----------FEAAEEIKNGFFGSLR-DPPLFNRDSSSSASMRR 164

Pt ----------GGINRF----------FEAAEEMKNNFFDVFG---DFHNGNSSSSPSKRR 156

Rc ----------GGIGRL----------FEAAEQMRSSIFYTFG---ELYKENSSPSPSR-- 167

Ca ----------GGLGRF----------FEAAEEMKNGFFDVIAKSPPIFDVESSSSSPMRR 156

Gm ----------SGLSHF----------FDAA----NGFFDVFSKSPSIFDAESSSPSVRR- 149

Vv ----------GGLNRF----------FEAAEEMKNGFFSVFG-APPVFDGESSSSSSTRR 160

Sl ----------SELDRF----------FEAAEEIKNGFFGAFS-IPRVFDDDRSPPPERR- 167

Pp DERQRSPAVPGGFTGFGGIMNAIEDVMRETEDMAHSFLHVFGLDGDETDRSGNPFDRWFG 201

. : . : : .:. :

Zm -------PADRHI-------EEDTA-----KNKTESDYSE-FRSKI-SDV 187

Sb -------PAEKHI-------EEDTA-----RKTKESDYSE-FRSKI-SDV 188

Os/Xb24 GRHIGGQPAGRHI-------EEGTA-----KDTKQNDYAE-FSSKI-TDV 198

Bd -------PTERHT-------EDGSS-----KKANENDYSN-FGSQI-TDV 186

At GI-----PIENHP-----KVEEHRNDETAPTRPHSSGEID-LSGLA-KDV 192

Al GI-----PIENHP-----KIEEHRNDKTAPTRPHSSGEID-LSGLA-KDV 192

Cs GI-----PIEEDH-----DPVFFYN----PKEPDS-GHVD-LSGLA-RDV 197

Pt GI-----PVEGHP-----LTEASPK----PKEPNS-GDVD-LSGLA-RDV 189

Rc GI-----PIEDYS-----RREASPK----ARETEP-GNVD-LSGLA-KDV 200

Ca GI-----PIEEYG-----RQETHPR----SKDMES-TDTD-FTALA-KDV 189

Gm GI-----PIEEYR-----RPEAYPK----SKEKES-GDTD-FVAMA-KDV 182

Vv GI-----PFEGSNP----TKEASAE----PFKAES-GYGD-LSGLA-REV 194

Sl GI-----PIESHP----------------PKPNNTDGDVN-ISGLD-NDI 194

Pp GGDVFGDSEKRAPR------RSEPD---GVHHPAQSSKPDIFDGRDFREV 242

. : : . ::
