# Supplementary material for: The Phylogenetically-Related Pattern Recognition Receptors EFR and XA21 Recruit Similar Immune Signaling Components in Monocots and Dicots
Source: PLoS Pathog. 2015 Jan 21;11(1):e1004602. doi: 10.1371/journal.ppat.1004602 (PMC4301810; doi:10.1371/journal.ppat.1004602)
Supplement: S6 Fig — Alignment of the XB15/POL homologues used to create the phylogenetic tree in Fig. 4D. Accession numbers are listed in S4 Table. (DOCX) [file ppat.1004602.s006.docx]

At2G28890/PLL4 ------------MGNGIGKLSKCLTGGAGRNKKP-ELSIL--------EPDPLDEGLGHS 39

At1G07630/PLL5 ------------MGNGVTKLSICFTGGGGERLRPKDISVL--------LPDPLDEGLGHS 40

At5G02400/PLL2 ------------MGNGVTTLTGCCTGTLAGEISR--RYD----------VSLVHDGLGHS 36

At3G09400/PLL3 ------------MGNGVASFSGCCAGTTAGEISG--RYVT--------GVGLVQENLGHS 38

Os03g0821300/XB15 ------------MGNSLACFCCGGGAGGRGGRHVAPAALP--------SDPAYDEGLGHS 40

At2G46920/POL ------------MGNGTSRVVGCFVP--SNDKNG--VDLE--------FLEPLDEGLGHS 36

Os03g0372500 MVLGLGVANQPAMGNSTSRVVGCFAP--ADKAAGGGVGLE--------FLQPLDEGLGHS 50

At2G35350/PLL1 ------------MGSGFSSLLPCFNQGHRNRRRHSSAANPSHSDLIDSFREPLDETLGHS 48

Os02g0690500 ------------MGSGASRLLTACTCSR--PAPASVDAEP-----------CLDDALGHS 35

Os03g0275100 ------------MGNGITKNP-CFSG---DPYAAAVASDP-----------LPDDSHGHS 33

Os04g0403701 -----------MVDEELFDKSSNDHSISSEEEDMLVRSYS-----------NLNVSFGYH 38

:.. . *:

At2G28890/PLL4 FCYVRPD---------PTR-----VSSSKVHSEEE------------------------- 60

At1G07630/PLL5 FCYVRPD---------PTL-----ISSSKVHSEEDT------------------------ 62

At5G02400/PLL2 FCYIRPD---------LPG-VVLPSPESPLRSDHIQ------------------------ 62

At3G09400/PLL3 FCYVRPV---------LTGSKSSFPPEPPLRPDPIPG----------------------- 66

Os03g0821300/XB15 FCYVRPDKFV------VPFSADDLVADAKAAAAAEG------------------------ 70

At2G46920/POL FCYVRP-SIFESPDITPSNSERFTIDSSTIDSETLTGSFRNDIVDDPSFLN------RHN 89

Os03g0372500 FCYVRPGAITDSPAITPSNSERYTLDSSVLDSETRSGSFRQEVVVVDDLAAAAMAGLQRP 110

At2G35350/PLL1 YCYVPSSSNR---FISPFPSDRFVSPTASFRLSPPHEPGRIR---------------GSG 90

Os02g0690500 FCYAAAAT--------------ATAHSSSFRHG--------------------------- 54

Os03g0275100 FTYVPSS---------------AAAFDHSPRSAAAS------------------------ 54

Os04g0403701 CNSYQCFS--------LDTDEYDISPNKRLETNTMMTS---------------------- 68

At2G28890/PLL4 -----TTTFRTISGASVSANTATP---------LSTSLYDPYGHIDR-----AAAFESTT 101

At1G07630/PLL5 ----TTTTFRTISGASVSANTATP---------LSTSLYDPYGHIDR-----AAAFESTT 104

At5G02400/PLL2 -----ETTFRSISGASVSANPSTA---------LSGALSSDSDCPYSS-AVSASAFESSG 107

At3G09400/PLL3 ----TTTTFRSISGASVSANTSTA---------LSTSLSTDTSG-------IASAFESSN 106

Os03g0821300/XB15 ----EATTFRAISGAALSANVSTP---------LSTSVLLLMPEESSASATASSGFESSE 117

At2G46920/POL SKGLAETTFKAISGASVSANVSTA---------RTGNQMALCSSDVLEP---AASFESTS 137

Os03g0372500 SKSFSETTFRTISGASVSANPSSA---------RTGNLCVSLAADVQEP---AAAFESTA 158

At2G35350/PLL1 SSEQLHTGFRAISGASVSANTSNSKTVLQLEDIYDDATESSFGGGVRRSVVNANGFEGTS 150

Os02g0690500 -----------ISGAALSANSSVP------VPLYN---ASAAAGGVAPGYSSA--FHTSS 92

Os03g0275100 ----SETSYFSLSGAAISANPATS----------ASMPSFRLYNELTWPPSTACTFESSR 100

Os04g0403701 ----QNGSFTCLSGAAISANFTLAN---------TNICKGLIGEEILP------ELDSPN 109

:***::*** : . :. .

At2G28890/PLL4 SFSSIPLQPIPRSS--------GPIVPGSGPLERG-FLSGPIER--GFMSGPLDGS---S 147

At1G07630/PLL5 SFSSIPLQPIPKSS--------GPIVLGSGPIERG-FLSGPIER--GFMSGPLDRVGLFS 153

At5G02400/PLL2 NFASLPLQPVPRGSTWQSGPIVNESGLGSAPFERR-FLSGPIES--GLYSGPIEST---K 161

At3G09400/PLL3 RFASLPLQPVPRSP-------IKKSDHGSGLFERR-FLSGPIES--GLVSGKKTKE---K 153

Os03g0821300/XB15 SFAAVPLQPVPRFS----------SGPISAPFSGG-FMSGPLER--GFQSGPLDAA---- 160

At2G46920/POL SFASIPLQPLPRGG------SGPLNGFMSGPLERG-FASGPLDRNNGFMSGPIEKG-VMS 189

Os03g0372500 SFAAVPLQPVPRG-------SGPLNTFLSGPLERG-FASGPLDKGAGFMSGPLDKGVFMS 210

At2G35350/PLL1 SFSALPLQPGPDRSG----------LFMSGPIERG-ATSGPLDPPAGEISRSNSAGVHFS 199

Os02g0690500 SFSSAPLQLSNLSSGP---------LFLSGPIDRAGQLSGPLDP-----------AVPFS 132

Os03g0275100 SFAAAPLIQAAPPR-----------LSMSGPLHAT---SGRFSEASGSASTASDRFS--D 144

Os04g0403701 SFRKIVSSP--------------------------------------------------- 118

*

At2G28890/PLL4 GPIDGKTGSD---QFQRSFSHGLANLRVGSRKGSLVRVLRRAISKTIT-RGQNSIVAPIK 203

At1G07630/PLL5 GPLD-KPNSDHHHQFQRSFSHGLA-LRVGSRKRSLVRILRRAISKTMS-RGQNSIVAPIK 210

At5G02400/PLL2 KTEKEKPK--------KIRKKPKS-KKN---FLTFKTLFANLISNNNKPRLKKSVIEPIN 209

At3G09400/PLL3 AKLKKSGS--------KSFTKPKL-KKSESKIFTFKNVFTNLSCS------KKSVIKPIN 198

Os03g0821300/XB15 -----------------LLSGPLPGTATSGRMGGAVPALRRSLSHGGR-RLRNFTRALLA 202

At2G46920/POL GPLDVSDR--------SNFSAPLSFRRKKPRFQRFMRSVSGPMKSTLA---RTFSRRSGG 238

Os03g0372500 GPIDSGNK--------SNFSAPLSYGRRKAGLGQLVRSISRPMRSALS---RTFSRSSQG 259

At2G35350/PLL1 APLGG--------------VYSKKRRKKKKKSLSWHPIFGGEKKQRPW------------ 233

Os02g0690500 GPLP-----------------AKPPKPASSSSRGFSRRFRKPS----------------- 158

Os03g0275100 HPFMDG-----------MLDRASSASSTARLMPSFSHLMSEPRVAQSGLSNERSLIRSLV 193

Os04g0403701 ---------------------------SMSRLDLLSTSQGSPVSTESS------------ 139

At2G28890/PLL4 PVKEPD-WVFGSDKTRIHQIENNLTVNSLNFSSEGSLLDDDV---S-------LESQNLQ 252

At1G07630/PLL5 SVKDSDNWGIRSEKSRNLHNEN-LTVNSLNFSSEVS-LDDDV---S-------LENQNLQ 258

At5G02400/PLL2 ---GSD----SSDSGRLHHEPVITSSRSNENPKSDLEEEDEK-QSM-------NSVLDVQ 254

At3G09400/PLL3 ---GFDSFDGSSDTDRYIPEINSLSTIVSSHEKPRIKEEEDKTESA-------LEEPKIQ 248

Os03g0821300/XB15 R------------TEKFQDSADLGSPDAAAAAVAACGGDPCG----------------LQ 234

At2G46920/POL ----------LSWMHRFFLHPETRVSWAVGKDGKLHGEDPESCLES---------NRNLQ 279

Os03g0372500 ----------TGWVQRFLLHPMAQLSLSR--DAKGTSEDSHNGLEAGLPELEYSVTRNLQ 307

At2G35350/PLL1 ----------VLPVSNFVVGAKKENIVRPDVEAMAASSGEND----------------LQ 267

Os02g0690500 ------------------FGSLRRSVSEKNRPCAVPLRRDDG----------------VQ 184

Os03g0275100 R---------VASKLRFGVPLSGRRSNGPAEPTTKSDGDYRSTPKG-----------NVE 233

Os04g0403701 ------------------IFEISKNIWRSSAPTTVSSNFLTS--------------TEIK 167

::

At2G28890/PLL4 WAQGKAGEDRVHVVVSEEHGWLFVGIYDGFNGPDAPDYLLSHLYPAVHRELKGLLWDD-- 310

At1G07630/PLL5 WAQGKAGEDRVHVVVSEEHGWLFVGIYDGFNGPDAPDYLLSHLYPVVHRELKGLLWDD-- 316

At5G02400/PLL2 WAQGKAGEDRVHVVVSEDNGWVFVGIYDGFSGPDAPDYLLNNLYTAVQKELNGLLWNDE- 313

At3G09400/PLL3 WAQGKAGEDRVHVILSEENGWLFVGIYDGFSGPDPPDYLIKNLYTAVLRELKGLLWID-- 306

Os03g0821300/XB15 WAQGKAGEDRVHVVVSEERGWVFVGIYDGFNGPDATDFLVSNLYAAVHRELRGLLWDQR- 293

At2G46920/POL WAHGKAGEDRVHVVLSEEQGWLFIGIYDGFSGPDAPDFVMSHLYKAIDKELEGLLWDYEE 339

Os03g0372500 WAHGKAGEDRVHVVLSEEQGWLFIGIYDGFSGPDAPDFLMSNLYKAIDKELEGLLWVYED 367

At2G35350/PLL1 WALGKAGEDRVQLAVFEKQGWLFAGIYDGFNGPDAPEFLMANLYRAVHSELQGLFWELEE 327

Os02g0690500 WAHGRAGEDRVHVVVSEDQRWLFVGIYDGFNGPEAPDFLVTNLYRFLLRELRGIFYKEAD 244

Os03g0275100 WAQGMAGEDRFHVAVSEEHGWVFVGIYDGFNGPDATDYLFANLYVAVHRELKGVLWDDIQ 293

Os04g0403701 MAGGAAGEDRVQAVCSEKNGWLICGIYDGFNGRDAADFLAVTLYDNIVYYLYLLECRIK- 226

* * *****.: *.. *:: ******.* :..::: ** : * :

At2G28890/PLL4 ------------------------------------------------------------

At1G07630/PLL5 ------------------------------------------------------------

At5G02400/PLL2 ------------------------------------------------------------

At3G09400/PLL3 ------------------------------------------------------------

Os03g0821300/XB15 ------------------------------------------------------------

At2G46920/POL PSED----------------------------------------------------NQLQ 347

Os03g0372500 SPEGSAQVSTLGEGESVAVPQDLPDGGD-----------------------ILFQADSVE 404

At2G35350/PLL1 EDDNPTDISTRELEQQGEFEDHVNEMASSSCPATEKEEEEMGKRLTSSLEVVEVKERKRL 387

Os02g0690500 ADN------------------------------------------------------KKL 250

Os03g0275100 G----------------------------------------------------------- 294

Os04g0403701 ------------------------------------------------------------

At2G28890/PLL4 -----------PKTDAKSSDEADVENRDS------------------------------- 328

At1G07630/PLL5 -----------SNVESKSQD-LERSNGDE------------------------------- 333

At5G02400/PLL2 -KLRSLGENGMTKTGKCSDEEDP-ESGKE------------------------------- 340

At3G09400/PLL3 -KGESYNRNGESNIEKQSTVEHASDSDQE------------------------------- 334

Os03g0821300/XB15 -EQNVQHDQRPDQPGSAPSTTASDNQDQW------------------------------- 321

At2G46920/POL PDQEPPTEENMCDPESISEQHSKSVVAES------------------------EEVMIDD 383

Os03g0372500 SEQLVNSEEQDVSNVKISDGGASQVQMDLNTSGQRDLVLQASSNQKLNAGEIVEEKVGAD 464

At2G35350/PLL1 WELLAEAQAED-ALDLSGSDRFAFSVDDA------------------------------- 415

Os02g0690500 WQFLVDGDDDDSELDFSGSGRFALSLD--------------------------------- 277

Os03g0275100 ---VDVVTDNLPDPALANATHLCFLDAGG------------------------------- 320

Os04g0403701 ------QENGLYGSPEGSLNGVKSELT--------------------------------- 247

At2G28890/PLL4 ------------------------------------------------------------

At1G07630/PLL5 ------------------------------------------------------------

At5G02400/PLL2 ------------------------------------------------------------

At3G09400/PLL3 ------------------------------------------------------------

Os03g0821300/XB15 ------------------------------------------------------------

At2G46920/POL ISS-------------LGNTDTQIADGPPGDS-----------AGPGKKSMRLYELLQLE 419

Os03g0372500 MGNNLQSTESYNSGRDISNTDVNTSFGCTSDVNTSTCCNEDVKSPKEIRSRRLFELLEME 524

At2G35350/PLL1 ------------------------------------------------------------

Os02g0690500 ------------------------------------------------------------

Os03g0275100 ------------------------------------------------------------

Os04g0403701 ------------------------------------------------------------

At2G28890/PLL4 ------------------------------------------------------------

At1G07630/PLL5 ------------------------------------------------------------

At5G02400/PLL2 ------------------------------------------------------------

At3G09400/PLL3 ------------------------------------------------------------

Os03g0821300/XB15 ------------------------------------------------------------

At2G46920/POL QWEGEEIGLKRYG-----GNVALNNMTNQVENPS----------TSGGGAGNDPCTTDRS 464

Os03g0372500 LLEEYNRNVSKLSPEGMKGRSIFNMQAGTTEESSRDIAELSRSSMAATGECLDDFENDKH 584

At2G35350/PLL1 ------------------------------------------------------------

Os02g0690500 ------------------------------------------------------------

Os03g0275100 ------------------------------------------------------------

Os04g0403701 ------------------------------------------------------------

At2G28890/PLL4 -------------------------SSEKKSKNW-----------EESQRR-----WRCE 347

At1G07630/PLL5 -------------------------SCSNQEKD------------ETCERW-----WRCE 351

At5G02400/PLL2 -------------------------NCPVINNDDAVASGAR---NQAKSLK-----WRCE 367

At3G09400/PLL3 -------------------------NCPVMNGND-VACGSRNITSDVKKLQ-----WRCE 363

Os03g0821300/XB15 -------------------------GRRRRTRRSRPPRG-----ADDDQRR-----WKCE 346

At2G46920/POL ALDG-----------------IPNSGQRHGTKKSQISSKIRRMYQKQKSLRKKLFPWSYD 507

Os03g0372500 SRSGDGVLGVDPKECNECSISSSSSGHKQILRRYLFGSKLRKMYKKQKLLQKKFFPWNYD 644

At2G35350/PLL1 --------------------IGAGNAVSVGSKRWLLLSKLKQGLSKQGISGRKLFPWKSG 455

Os02g0690500 --------------------------------------RLKE---------SRFHMWAHA 290

Os03g0275100 --------------------------------------------------------VGGG 324

Os04g0403701 --------------------------------------------------------LAMR 251

At2G28890/PLL4 WDR-----DLDRLLKDR-SNGLDL--DPDPNSSDVLKALSQALRKTEEAYLENADMMLDE 399

At1G07630/PLL5 WDRES--QDLDRRLKEQISRRSGS--DRLTNHSEVLEALSQALRKTEEAYLDTADKMLDE 407

At5G02400/PLL2 WEKKS---NNKTKSDNRCDQKGSN--STTTNHKDVLKALLQALRKTEDAYLELADQMVKE 422

At3G09400/PLL3 WEHNS---SNK---------------SNNINHKDVLRALQQALEKTEES----FDLMVNE 401

Os03g0821300/XB15 WEQER----DCSNLKPPTQQRLRC--NSENDHVAVLKALTRALHRTEEAYLDIADKMVGE 400

At2G46920/POL WHREEGICVEEKIVESSGPIRRRW--SGTVDHDAVLRAMARALESTEEAYMDMVEKSLDI 565

Os03g0372500 WHRDQ-PHVDESVIKPSEVTRRCK--SGPVDHDAVLRAMSRALENTEEAYMDVVERELDK 701

At2G35350/PLL1 VEENETEEVDNVGVEEGVDKRRKRRKAGTVDHELVLKAMSNGLEATEQAFLEMTDKVLET 515

Os02g0690500 AADESGREWG---------SRRLAPAPAVRDHAAVLAALTRALASTEAAYLDMTDQSMGT 341

Os03g0275100 GDDDP---DAERKAKRGRIERNADDDGASSVHRDVLKALARALARTEEAFFAAAEERAAQ 381

Os04g0403701 FAENE------------------DVKFSETFRAGVLKCLTTAVEQAENDFLCMVEQEMDD 293

** .: .: :* :

At2G28890/PLL4 NPELALMGSCVLVMLMKGEDVYLMNVGDSRAVLGQKAESDYWI----------------- 442

At1G07630/PLL5 NPELALMGSCVLVMLMKGEDIYVMNVGDSRAVLGQKSEPDYWL----------------- 450

At5G02400/PLL2 NPELALMGSCVLVTLMKGEDVYVMNVGDSRAVLGRKPNLATG------------------ 464

At3G09400/PLL3 NPELALMGSCVLVTLMKGEDVYVMSVGDSRAVLARRPNVEK------------------- 442

Os03g0821300/XB15 FPELALMGSCVLAMLMKGEDMYIMNVGDSRAVLATMD----------------------- 437

At2G46920/POL NPELALMGSCVLVMLMKDQDVYVMNVGDSRAILAQERLHDRHSNPGFGNDEGIGHKSRSR 625

Os03g0372500 NPELALMGSCVLVMLMKDQDVYVMNLGDSRVVLAQD--NEQYNNSSFLKG-DLRHRNRSR 758

At2G35350/PLL1 NPELALMGSCLLVALMRDDDVYIMNIGDSRALVAQYQVEET------------------- 556

Os02g0690500 HPELAVTGACLLVALVRDDNVYVMNLGDSRAIVAQRPDDGDD------------------ 383

Os03g0275100 SPELGLVGSCVLVMLMKGKDVYLMNVGDSRAVLARRREPD-------------------- 421

Os04g0403701 RPDLVSVGSCVLVVLLHGTDLCILNLGDSRAVLASVPSSGMD------------------ 335

*:* *:*:*. *::. :: ::.:****.::.

At2G28890/PLL4 -GKIKQDLERINEETMN-DFDGCGDGEGAS-------------LVPTLSAFQLTVDHSTN 487

At1G07630/PLL5 -AKIRQDLERINEETMMNDLEGC-EGDQSS-------------LVPNLSAFQLTVDHSTN 495

At5G02400/PLL2 -RKRQKELERIREDSSLEDKEILMNGAMR----------------NTLVPLQLNMEHSTR 507

At3G09400/PLL3 -MKMQKELERVKEESPLETLFITERGLS------------------LLVPVQLNKEHSTS 483

Os03g0821300/XB15 ----SVDLEQISQ--------GSFDGSVGD-------------CPPCLSAVQLTSDHSTS 472

At2G46920/POL ESLVRIELDRISEESPIHNQATPISVSNKNR--------DVTSYRLKMRAVQLSSDHSTS 677

Os03g0372500 ESLVRVELDRISEESPMHNPNSHLSSNTKTK--------ELTICKLKMRAVQLSTDHSTS 810

At2G35350/PLL1 -GESVETAERVEERRNDLDRDDGNKEPLVVDSSDSTVNNEAPLPQTKLVALQLTTDHSTS 615

Os02g0690500 -GCVFGTMRRMEDVGVGLEIETRPGGCAIIG----------------LKPLQLSTDHSTS 426

Os03g0275100 ---FKDIFFRPDQDLQLLKAEVMRELEAHDR--------------NGLQCVQLTPEHSAA 464

Os04g0403701 ----------------------------------------------KLKAVQLTEIHSLE 349

: .**. **

At2G28890/PLL4 VEEEVNRIRKEHPDDASAVSNERVKGSLKVTRAFGAGFLKQP------------------ 529

At1G07630/PLL5 IEEEVERIRNEHPDDVTAVTNERVKGSLKVTRAFGAGFLKQP------------------ 537

At5G02400/PLL2 IEEEVRRIKKEHPDDDCAVENDRVKGYLKVTRAFGAGFLKQP------------------ 549

At3G09400/PLL3 VEEEVRRIKKEHPDDILAIENNRVKGYLKVTRAFGAGFLKQP------------------ 525

Os03g0821300/XB15 VEEEVIRIRNEHPDDPSAISKDRVKGSLKVTRAFGAGFLKQP------------------ 514

At2G46920/POL VEEEIWRIRSEHPEDDQSILKDRVKGQLKVTRAFGAGFLKKP------------------ 719

Os03g0372500 VEEEVSRIRAEHPDDPQSVFNDRVKGQLKVTRAFGAGFLKKP------------------ 852

At2G35350/PLL1 IEDEVTRIKNEHPDDNHCIVNDRVKGRLKVTRAFGAGFLKQP------------------ 657

Os02g0690500 IEEEVHRIKREHPDDDQCIVNDRVKGRLKVTRAFGAGYLKQA------------------ 468

Os03g0275100 AEEEVRRIRSQHLTDRQAVVNGRVKGKLSVTRAFGAGYLKQP------------------ 506

Os04g0403701 NPLEYQKLLADHPNEPSVVMGNKIKGKLKVTRAFGVGYLKQVTYYNFVWEERALMHLCSY 409

* :: :* : : ::** *.******.*:**:

At2G28890/PLL4 --------------KWNNALLEMFQIDYKGTSPYINCLPSLYHHRLGSKDQFLILSSDGL 575

At1G07630/PLL5 --------------KWNNALLEMFQIDYVGKSPYINCLPSLYHHRLGSKDRFLILSSDGL 583

At5G02400/PLL2 --------------KWNDALLEMFRIDYIGTSPYITCSPSLCHHKLTSRDKFLILSSDGL 595

At3G09400/PLL3 --------------KWNEALLEMFRIDYVGTSPYITCSPSLHHHRLSSRDKFLILSSDGL 571

Os03g0821300/XB15 --------------KWNDALLEMFRIDYVGSSPYISCNPSLFHHKLSTRDRFLILSSDGL 560

At2G46920/POL --------------NFNEALLEMFQVEYIGTDPYITCEPCTVHHRLTSSDRFMVLSSDGL 765

Os03g0372500 --------------KFNDILLEMFRIDYVGTSSYISCNPAVLHHRLCSNDRFLVLSSDGL 898

At2G35350/PLL1 --------------KLNDALLEMFRNEYIGTDPYISCTPSLRHYRLTENDQFMVLSSDGL 703

Os02g0690500 --------------KLNNGLLEMFRNDYIGDTPYISCTPSLCHHKLTARDQFLVLSSDGL 514

Os03g0275100 --------------KWNDRLLEAFKVDYIGAEPYISCTPSLRHHRISSNDRFLVLSSDGL 552

Os04g0403701 DFTNTHALHVNLQKKLNDALMGILRVRNLCSPPYVYTNPHTVSHKVTEDDLFVVLGSDGL 469

: *: *: :: .*: * ::: * *::*.****

At2G28890/PLL4 YQYFTNEEAVSEVELFITLQPEGDPAQHLVQELLFRAAKKAGMDFHELLEIPQGERRRYH 635

At1G07630/PLL5 YQYFTNEEAVSEVELFITLQPEGDPAQHLVQELLFRAAKKAGMDFHELLEIPQGERRRYH 643

At5G02400/PLL2 YEYFSNQEAIFEVESFISAFPEGDPAQHLIQEVLLRAANKFGMDFHELLEIPQGDRRRYH 655

At3G09400/PLL3 YEYFSNEEAIFEVDSFISAFPEGDPAQHLIQEVLLRAAKKYGMDFHELLEIPQGDRRRYH 631

Os03g0821300/XB15 YQYFTNEEAVAQVEMFIATTPEGDPAQHLVEEVLFRAANKAGMDFHELIEIPHGDRRRYH 620

At2G46920/POL YEYFSNEEVVAHVTWFIENVPEGDPAQYLIAELLSRAATKNGMEFHDLLDIPQGDRRKYH 825

Os03g0372500 YQYFSNDEVVSHVAWFMENVPEGDPAQYLVAELLCRAAKKNGMDFHELLDIPQGDRRKYH 958

At2G35350/PLL1 YQYLSNVEVVS---LAMEKFPDGDPAQHVIQELLVRAAKKAGMDFHELLDIPQGDRRKYH 760

Os02g0690500 YQYLSNEEVVLHVENFMERFPEGDPAQSLIEELLSRAAKKAGMDFYELLDIPQGDRRKYH 574

Os03g0275100 YQYFTNKEVVDQVAMFTAEQPDGDPAKHLVGELVLRAARKAGMDCRRLLEIPHGDRRNYH 612

Os04g0403701 FDFFSNDEVVQLVYQFMHDNPIGDPAKYLIEQLLLKAAKEAALTAEELMRIPVGSRRKYH 529

:::::* *.: * ****: :: ::: :** : .: *: ** *.**.**

At2G28890/PLL4 DDVSIVVISLEG-RMWKSCV------------ 654

At1G07630/PLL5 DDVSIVVISLEG-RMWKSCV------------ 662

At5G02400/PLL2 DDVSVIVISLEG-RIWRSSM------------ 674

At3G09400/PLL3 DDVSVIVISLEG-RIWRSSM------------ 650

Os03g0821300/XB15 DDVSVIVISLEG-RIWRSCV------------ 639

At2G46920/POL DDVSVMVVSLEG-RIWRSSGQYYPERKQKFNR 856

Os03g0372500 DDVSVMVISLEG-RIWRSSG------------ 977

At2G35350/PLL1 DDCTVLVIALGGSRIWKSSGKYL--------- 783

Os02g0690500 DDVTVMVISLEG-RIWKSSGTYV--------- 596

Os03g0275100 DDVSIIVMSFEG-RIWRSSV------------ 631

Os04g0403701 DDVTIIVIILGNAQRTMTASTSL--------- 552

** :::*: : . : :.
